# The regulation of the sulfur amino acid biosynthetic pathway in *Cryptococcus neoformans*: the relationship of Cys3, Calcineurin, and Gpp2 phosphatases

**DOI:** 10.1038/s41598-019-48433-5

**Published:** 2019-08-15

**Authors:** Amanda Teixeira de Melo, Kevin Felipe Martho, Thiago Nunes Roberto, Erika S. Nishiduka, Joel Machado, Otávio J. B. Brustolini, Alexandre K. Tashima, Ana Tereza Vasconcelos, Marcelo A. Vallim, Renata C. Pascon

**Affiliations:** 1 Universidade Federal de São Paulo, Campus Diadema, São Paulo, SP Brazil; 2 Departamento de Bioquímica, Escola Paulista de Medicina, Universidade Federal de São Paulo Campus São Paulo, São Paulo, SP Brazil; 3 Laboratório Nacional de Computação Científica- LNCC, Labinfo- Laboratório de Bioinformática, Petrópolis, Rio de Janeiro, Brazil

**Keywords:** Fungal pathogenesis, Pathogens

## Abstract

Cryptococcosis is a fungal disease caused by *C*. *neoformans*. To adapt and survive in diverse ecological niches, including the animal host, this opportunistic pathogen relies on its ability to uptake nutrients, such as carbon, nitrogen, iron, phosphate, sulfur, and amino acids. Genetic circuits play a role in the response to environmental changes, modulating gene expression and adjusting the microbial metabolism to the nutrients available for the best energy usage and survival. We studied the sulfur amino acid biosynthesis and its implications on *C*. *neoformans* biology and virulence. CNAG_04798 encodes a BZip protein and was annotated as *CYS3*, which has been considered an essential gene. However, we demonstrated that *CYS3* is not essential, in fact, its knockout led to sulfur amino acids auxotroph. Western blots and fluorescence microscopy indicated that GFP-Cys3, which is expressed from a constitutive promoter, localizes to the nucleus in rich medium (YEPD); the addition of methionine and cysteine as sole nitrogen source (SD–N + Met/Cys) led to reduced nuclear localization and protein degradation. By proteomics, we identified and confirmed physical interaction among Gpp2, Cna1, Cnb1 and GFP-Cys3. Deletion of the calcineurin and *GPP2* genes in a GFP-Cys3 background demonstrated that calcineurin is required to maintain Cys3 high protein levels in YEPD and that deletion of *GPP2* causes GFP-Cys3 to persist in the presence of sulfur amino acids. Global transcriptional profile of mutant and wild type by RNAseq revealed that Cys3 controls all branches of the sulfur amino acid biosynthesis, and sulfur starvation leads to induction of several amino acid biosynthetic routes. In addition, we found that Cys3 is required for virulence in *Galleria mellonella* animal model.

## Introduction

*C*. *neoformans* is an opportunistic yeast that causes serious disease in immunocompromised patients^1^. This pathogen is widely distributed in nature; it can be encountered in wood, bird excreta, soil, animals, and in the human host^2–4^. The broad occurrence implies that *C*. *neoformans* is able to adapt to different ecological niches. This plasticity reflects metabolic flexibility and tight genetic regulation of gene expression allowing fast adaptation to sudden environmental changes, facilitating survival and persistence in the host^5–7^. In immunocompromised patients that cannot properly fight infectious agents, *C*. *neoformans* finds the perfect circumstances to invade and cause meningoencephalitis, a difficult condition to treat^8,9^. Besides its clinical relevance, *C*. *neoformans* has been extensively used for basic studies, since it is a malleable model organism to study virulence and the molecular bases of host and pathogen interactions^3,10^.

**Figure 1:**
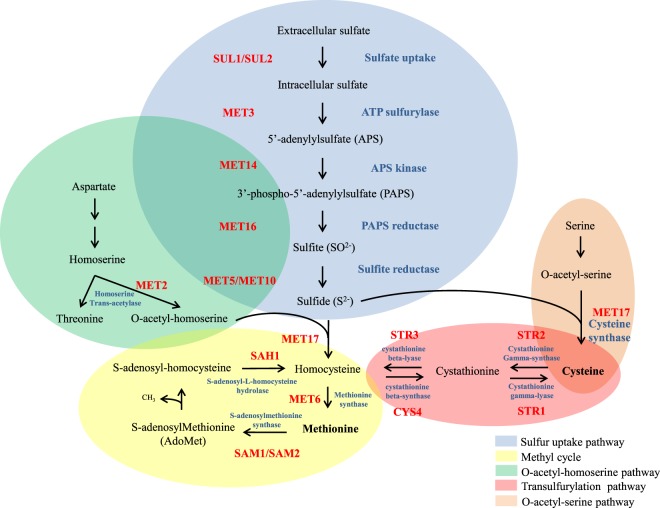
Sulfur uptake and sulfur amino acid biosynthesis pathway proposed for *C*. *neoformans*. Based on *S*. *cerevisiae* and *N*. *crassa* pathways^32^.

Adaptation to various nutritional conditions is a major trait to pathogen establishment in the host and disease progression^6,11,12^. Our previous work and others have shown that amino acid biosynthesis and uptake directly impact virulence and may be relevant as drug targets^13–21^. Sulfur uptake and methionine biosynthetic pathway have been explored and found to modulate *C*. *neoformans* survival in the host^21–23^. Sulfur is especially important for all living organisms because protein synthesis initiation depends on methionine, and cysteine is essential for disulfide bonds which maintain protein structure and function. S-adenosyl methionine (SAM) also plays an important role for methyl group transfer as well as lipid and polyamine biosynthesis^24–28^. As a core nutrient, sulfur uptake must be under a very tight regulatory network, which guarantees the best use of its supply. In *Neurospora crassa*, *Aspergillus nidulans*, and *Saccharomyces cerevisiae*, a set of transcriptional regulators, permeases, and catalytic enzymes involved in sulfur uptake are well known^27,29–32^. Figure 1 presents the sulfur uptake coupled to sulfur amino acid biosynthetic pathway, which starts with intracellular transport of inorganic sulfate by permeases. In *N*. *crassa* and *S*. *cerevisiae*, two genes encode the sulfate permeases: *cys-13* and *cys-14* and *SUL1* and *SUL2*, respectively^33,34^. Once sulfate is internalized, the first step in assimilation is carried out by ATP sulfurylase (*MET3*), yielding adenosine phosphosulfate (APS), which, in turn, is phosphorylated by APS Kinase (*MET14* in *S*. *cerevisiae*), producing 3´-phosphoadenosine-5´-phosphosulfate (PAPS), a key intermediate in sulfurylation pathway. From this point on, PAPS reductase (*MET16*) and sulfite reductase (*MET5* and *MET10*) catalyze the production of sulfide. The major cysteine biosynthesis pathway is carried out by cysteine synthase (*MET17*), which promotes the condensation of sulfide and O-acetyl-serine into cysteine (Fig. 1). Homocysteine can also be synthesized by *MET17* from O-acetyl-homoserine and sulfide. Homocysteine gives rise to cysteine in the transulfurylation pathway and vice-versa and methionine is synthesized from homocysteine through *MET6* (methionine synthase). Homocysteine can also be generated through the methyl cycle, making it an important intermediary metabolite that is substrate for the biosynthesis of both sulfur amino acids^27,32,35–40^.

In *N*. *crassa*, three gene encoding regulators of the structural genes that act in this branched pathway have been thoroughly studied: *scon-1*, *scon-2*, and *cys-3*^29,30,41–52^. *A*. *nidulans* also encodes *scon1*, *scon2*, and *metR*, which is the equivalent of *N*. *crassa* c*ys-*3. *metZ*, a second positive regulator has been described and also plays a role on sulfur uptake^31,32,53^. The first two proteins function as negative regulators of *cys-3/metR*, the major BZip transcription factor that activates the transcription of the sulfate permeases *cys-13* and *cys-14* and other structural enzymes involved in sulfate assimilation. The inactivation of these negative factors leads to over expression of *cys-3* and its targets^32^. In *S*. *cerevisiae*, the regulation of the sulfur uptake is different. With a *Cys*-3 homologue encoded by *MET4*, which is a transcriptional activator that has no ability to bind DNA, *S*. *cerevisiae* relies on DNA-binding factors *CBF1* and *MET31/MET32* and a co-factor (*MET28*) that stabilizes the complex, to promote transcription of the structural enzymes involved in the sulfur uptake network^27,54–56^. In *N*. *crassa*, *A*. *nidulans*, and *S*. *cerevisiae*, *cys*-3/*MET4* expression is induced in response to sulfur limitation, and the protein quickly disappears in the presence of methionine and cysteine and, in some cases, S-adenosyltmethionine (AdoMet)^29,30,32^. Experimental data in *S*. *cerevisiae* indicated that *Scon*-2/*MET30* encodes the substrate recognition subunit of the ubiquitin ligase and is responsible for *MET4* degradation in the presence of cysteine, but not methionine or AdoMet, which provides a mechanistic explanation for how the sulfur uptake is genetic regulated upon a nutritional signal^27^. *MET4* degradation may also be triggered by oxidative stress in *S*. *cerevisiae* and mutations that affect sulfur metabolism in *A*. *nidulans* induced environmental stress responses, suggesting a probable link between sulfur amino acid biosynthesis and stress response or adaptation^57,58^.

Historically, the main genetic players of the sulfur uptake network have been identified in *N*. *crassa*, *A*. *nidulans*, and *S*. *cerevisiae*^32^. Elements of the sulfur regulatory pathway have been uncovered in *Alternaria alternate*, *Aspergillus fumigatus*, and *Mycobacterium*. The first is considered a plant pathogen and the last two, human pathogens^59–61^. The deletion of *metR* from *A*. *alternate* and *A*. *fumigatus* led to decrease virulence in both pathogens, which is consistent with previous literature published, in which *C*. *neoformans met3Δ* and *met6Δ* mutants are avirulent in animal model, reinforcing the idea that sulfur assimilation maybe an important feature for fungal pathogenesis^22,23,59,61,62^. Despite the clear connection among sulfur assimilation, sulfur amino acid biosynthesis, and virulence, very little is known about the interrelation of these biological processes and their regulatory mechanisms in *C*. *neoformans*. Moreover, from what we have learned from *N*. *crassa*, *A*. *nidulans*, and *S*. *cerevisiae*, the genetic regulation of sulfur uptake may be different among diverse organisms.

*In silico* analysis revealed that *C*. *neoformans* genome encodes a BZip protein annotated as *CYS3* (CNAG_04798), which has been considered an essential gene^63^. However, our data show that, on the contrary, *CYS3* deletion is not lethal, but renders cells auxotrophic for sulfur amino acids. *CYS3* knockout strains accumulate stress sensitivities and have attenuated virulence in animal model. In addition, the expressed GFP-Cys3 localizes to the nucleus in complex medium, presumably, a sulfur limiting condition, but this fusion protein is degraded upon sulfur amino acid supplementation.

Searches for sequence similarities using bioinformatics tools revealed that *C*. *neoformans* does not encode homologues of *S*. *cerevisiae MET31*, *MET32*, or *MET28*; however, there is a *MET30* F-Box protein with the typical WD 40 repeats (CNAG_05774), which in *S*. *cerevisiae* and *N*. *crassa* is known to act upon *MET4*/*cys-3* triggering its degradation in response to sulfur amino acid supplementation^64^. This suggests that, while *C*. *neoformans* has part of the genetic elements that control sulfur assimilation, the regulatory network may be different from other fungi. Therefore, proteomics approaches were applied to identify Cys3 partners in hope of uncovering the sulfur regulatory network. A GFP-Cys3 fusion protein was immunoprecipitated and mass spectrometry was applied to discover a putative protein complex. Several proteins were identified in association to Cys3, and two of them were further analyzed: the calcineurin (catalytic and regulatory subunits, Cna1 and Cnb1, respectively) and a protein phosphatase (Gpp2) involved in glycerol biosynthesis, response to hyperosmotic and oxidative stress, and during diauxic shift in *S*. *cerevisiae*^65–67^. Yeast two hybrid assays demonstrated that these proteins physically interact. Further, GFP-Cys3 cellular localization and abundance are dependent on calcineurin and Gpp2. Global transcriptional profile of *cys3Δ* mutant by RNAseq revealed interesting features of the amino acid biosynthetic response under sulfur amino acid starvation. Our investigation on Cys3 transcription factor has brought to light novel insights into the regulatory mechanism of sulfur amino acid biosynthesis, an important nutritional aspect of *C*. *neoformans* that deeply impacts virulence.

## Results

### The role of Cys3 transcription factor in the biosynthesis of sulfur amino acids in *C*. *neoformans*

To find genetic elements controlling sulfur amino acid biosynthesis, we studied a gene annotated as *CYS3*. CNAG_04798 encodes a protein containing a conserved BZip domain, a nuclear localization signal, followed by a leucine zipper (Supplementary Fig. 1). In *N*. *crassa* and *A*. *nidulans*, *cys-3* encodes a major BZip transcriptional factor involved in sulfur uptake; therefore, its deletion is expected to affect the methionine and cysteine biosynthesis in *C*. *neoformans*^29,31^. However, in an attempt to profile the transcription factor network, Jung *et al*. (2015) systematically deleted most of the transcription factors in *C*. *neoformans*^63^. In their report, *CYS3* was considered an essential gene^63^. This result was consistent with our gene deletion experiments to knockout *CYS3* in H99 strain, which turned out to be unsuccessful (89 transformants screened in YEPD + G418 on 9 different biolistic transformation experiments), which reinforced the idea that *CYS3* is essential.

Our previous work suggests that YEPD may not have enough amino acids to supplement some auxotrophs^14^. Hence, following biolistic transformation with a *cys-3Δ*::*Neo*^R^ linear PCR product, transformants were selected on SD supplemented with G418, amino acids (methionine, cysteine, and serine), and proline as the main nitrogen source. By supplying exogenous sulfur amino acids and relieving the nitrogen catabolite repression on permeases (proline), we were able to select two transformants containing homologous integration of the deletion cassette at the *CYS3 locus* (CNU123 and CNU124), as shown by diagnostic PCR and Southern blot (Supplementary Fig. 2). A complemented strain was also generated by transforming CNU123 with a linear PCR product containing the wild type allele of *CYS3* gene and pZPHyg harboring the hygromycin resistance cassette. We applied qPCR to quantify the *CYS3* expression levels in the knockout and the complemented strains relative to wild type. The results found no detectable *CYS3* expression in the knockout mutants and intermediary levels of *CYS3* expression in all 3 complemented strains relative to wild type (Supplementary Fig. 3). Based on this result, CNU136 was selected as the complemented strain used for further analysis.

**Figure 2:**
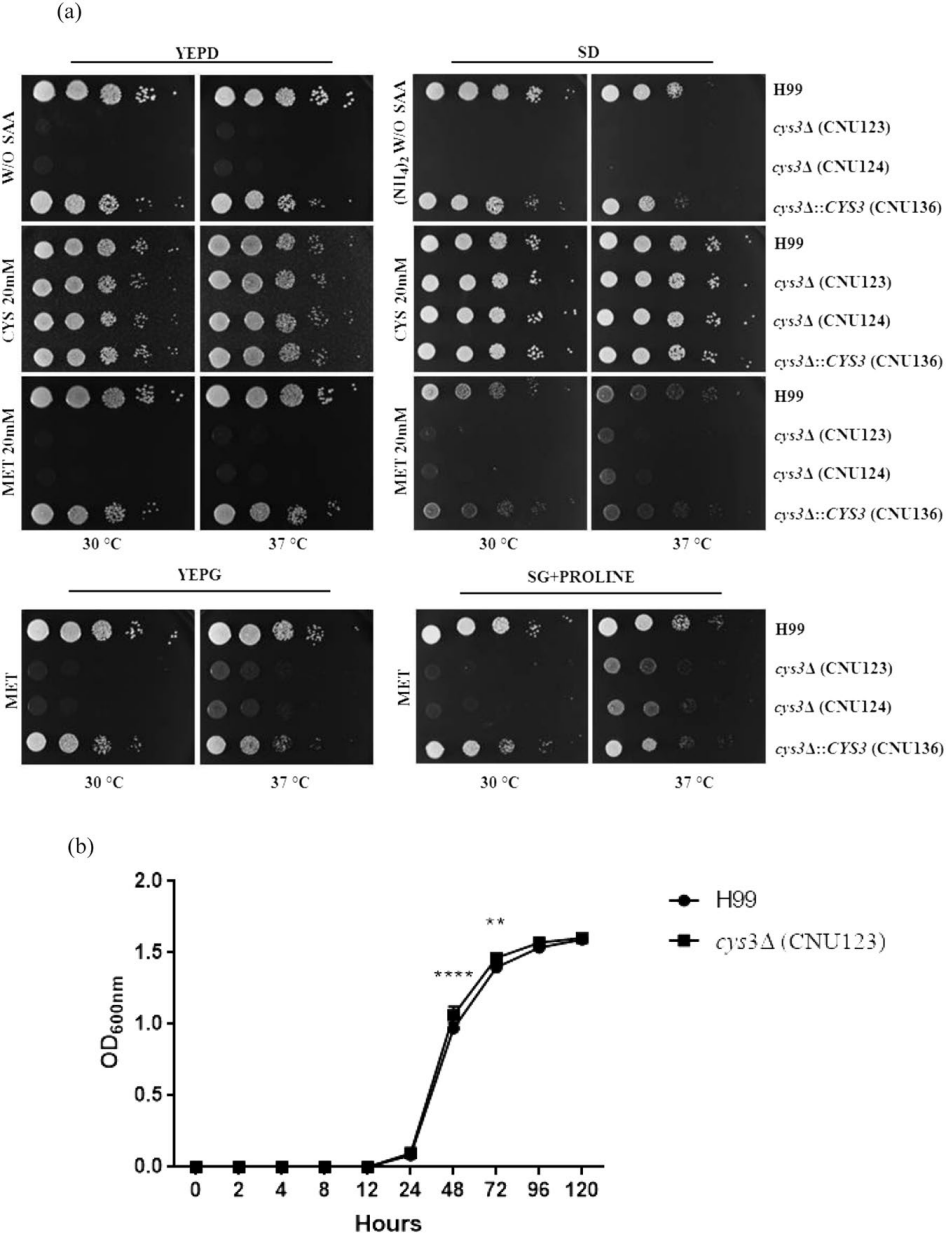
(**a**) Growth pattern by spot dilution of wild type (H99), *cys3Δ* mutants (CNU123 and CNU124), and complemented strain (CNU136) in YEPD and SD (synthetic dextrose) without sulfur amino acids (SAA), with 20 mM of cysteine (Cys) and 20 mM of methionine (Met) in two different temperatures (30 and 37 °C). The last rows show the strains in YEPG (rich medium added with galactose) and 20 mM methionine and synthetic galactose (SG) with proline as nitrogen source; (**b**) growth pattern evaluated by optical density at 600 ηm (OD_600_) of wild type (H99) and *cys3Δ* mutant (CNU123) in YEPD supplemented with 25 mM homocysteine for 120 h at 30 °C. Asterisks represent values that are statistically significant between wild type and mutant at a given time points (*p < 0.05; **p < 0.01, and ***p < 0.001).

The phenotypic analysis of the wild type, mutant, and complemented strains confirmed that *cys3Δ* is an auxotroph, requiring exogenous supplementation of sulfur amino acids to grow (Fig. 2a). On YEPD, the growth was mostly restored by supplementation with 20 mM cysteine. Increasing concentrations of methionine and cysteine did not improve growth. The addition of 20 mM cysteine restored growth to wild type levels in SD in both temperatures, 30 °C and 37 °C (Fig. 2a). Methionine did not restored growth on YEPD but promoted a slight increment in growth rate in SD at 37 °C (Fig. 2a). The best growth rate on methionine was obtained in the presence of non preferred carbon (galactose) and nitrogen (proline) sources (Fig. 2a), a nutritional condition which is known to induce amino acid permease gene expression and favors the uptake of methionine^14,15^; however, even in this condition, growth was considered poor (Fig. 2a). Homocysteine is a central intermediary metabolite in sulfur amino acid biosynthesis that should be able to satisfy methionine and cysteine auxotrophs (Fig. 1). Growth was restored to wild type levels in YEPD supplemented with 25 mM homocysteine (Fig. 2b).

**Figure 3:**
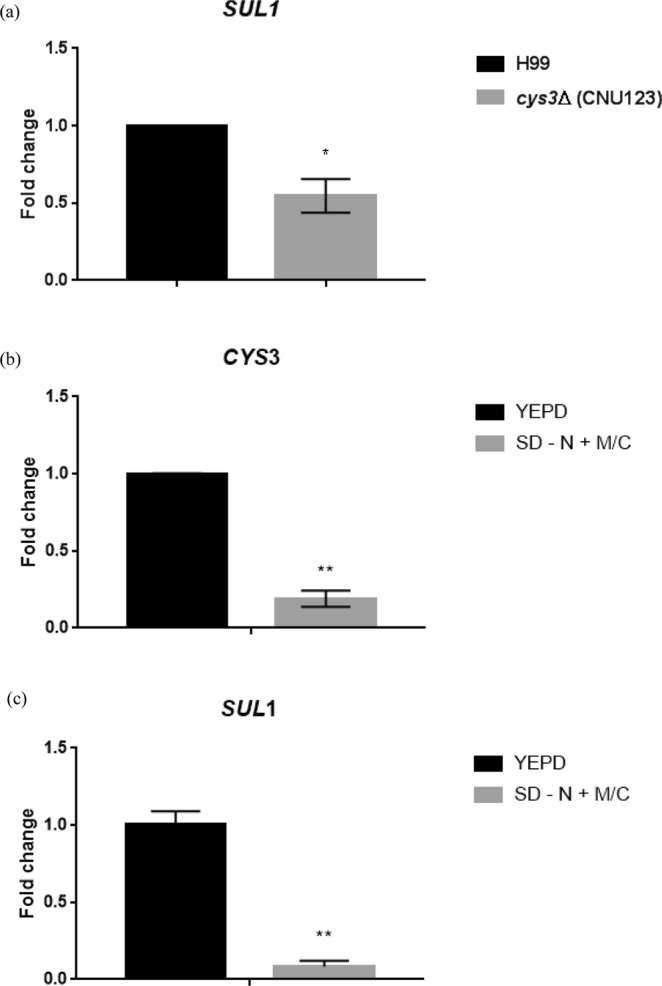
Expression pattern obtained by qPCR of (**a**) *C*. *neoformans* sulfate permease (*SUL1*) in wild type (H99) and *cys3Δ* (CNU123) in YEPD; (**b**,**c**) expression profile of *CYS3* and *SUL1*, respectively, in two different nutritional conditions (YEPD and SD - N + MC). Asterisks represent statistically different values at *p < 0.05 and ***p < 0.001.

These conclusions motivated us to further investigate how the sulfur network is activated in *C*. *neoformans* according to the type of medium supplied. To address this issue, the expression level of *CYS3* and the sulfate permease (*SUL1*) was evaluated in YEPD and SD added with sulfur amino acids. *SUL1* has not been described in *C*. *neoformans*, to the best of our knowledge. A bioinformatics search identified a gene (CNAG_00077) encoding a protein with high sequence similarity to *S*. *cerevisiae* sulfate permeases *SUL1* and *SUL2*^34,68^. In *A*. *nidulans* and *N*. *crassa*, the sulfate permeases expression is controlled by C*ys*-3^29^; therefore, we evaluated the expression levels of *SUL1* in wild type and *cys3Δ*::*Neo*^R^ (CNU123). We also analyzed the expression of the sulfate permease in complex and SD+ Met/Cys media by qPCR. *SUL1* expression in the mutant is 54% reduced compared to the wild type (YEPD), indicating that sulfate permease encoded by CNAG_00077 is, at least partially, controlled by Cys3 (Fig. 3a); therefore, it is likely part of the sulfate uptake pathway. In the wild type strain (H99), *CYS3* and *SUL1* are both highly repressed in sulfur amino acids containing medium relative to YEPD (Fig. 3b,c). Our interpretation of this data is that complex medium is a sulfur limiting condition (low sulfate and sulfur amino acids), probably leading to induction of the sulfate uptake pathway. On the other hand, sulfur amino acids added to the medium serve as excellent sulfur source dispensing *de novo* sulfur amino acid biosynthesis.

Taken together, these results suggest that in *C*. *neoformans* (i) *CYS3* is involved in the sulfur uptake pathway, which seems to be a limited nutrient in complex medium (YEPD); (ii) cysteine is a better sulfur source than methionine, even though nutritional conditions that favor amino acid uptake can improve growth on methionine, probably by enhancing amino acid permease expression; (iii) *C*. *neoformans* has a transulfurylation pathway capable of synthesizing methionine from cysteine; however, (iv) methionine does not seem to be efficiently converted into cysteine, since methionine, as a sole sulfur source promotes only poor growth, suggesting that, even though transulfurylation pathway is present in *C*. *neoformans*, the methyl cycle may not be efficient in regenerating homocysteine in the *CYS3* deletion strain; (v) *SUL1* and *CYS3* are highly induced in complex medium and repressed in Met/Cys, suggesting that complex medium is a sulfur limiting medium for *C*. *neoformans* and supplementation with Met/Cys, the end products of the sulfur uptake network, signals the inhibition of the sulfur uptake pathway.

### The role of *CYS3* in *C*. *neoformans* virulence

**Figure 4:**
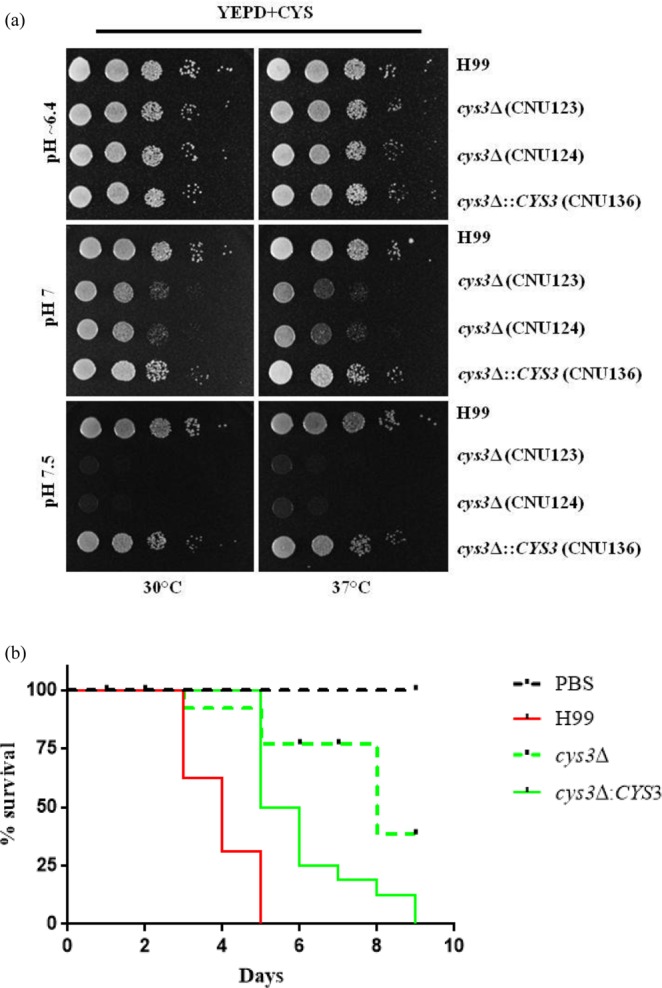
(**a**) Growth evaluation of wild type (H99), *cys3* mutants (CNU123 and CNU124), and complemented strain (CNU136) by spot dilutions on YEPD added with 20 mM cysteine in different pHs (6.4 and 7.0 and 7.5); (**b**) virulence assay in *G*. *mellonella* with H99 as positive control, PBS as negative and test strains *cys3Δ* (CNU123), and complemented strain (CNU136). Virulence assay expressed as percentage of larvae survival during 10 days at 30 °C. There are statistical significant differences among the survival curves at a *p* < 0,001 (***).

Wild type (H99), two *cys3Δ*::*Neo*^R^ deletion strains (CNU123 and CNU124) and the complemented strain (CNU136) were submitted to saline (0.75 M and 1 M NaCl), osmotic (0.75 M and 1 M KCl), oxidative stresses (1.5 and 3 mM H_2_O_2_) and cell membrane stress (SDS and Triton). All experiments were done in the presence of 20 mM cysteine. The growth rate was the same in mutant, complement, and wild type strains, suggesting that Cys3 does not affect the response to these stresses. The exposure of the same strains to cell wall disrupting agent, Congo red, led to growth arrest of the mutant (CNU123 and CNU124) at 0.25 and 0.5%. However, 4 different concentrations of calcofluor white (0.5, 1, 3 and 5 mg/mL) caused no effect in any of the strains tested; suggesting that *cys3Δ* mutant is not sensitive to cell wall stressor. The addition of Congo red to the medium may interfere with cysteine uptake leading to low growth. When alkaline stress was tested at 30 and 37 °C, the mutant strains grew better at the usual pH 6.5 in YEPD (Fig. 4a), as pH increased to 7.0 and 7.5 the growth of the mutants was progressively lower. However, this may be an effect of improved amino acids transport across the membrane at lower pH, since APC-like permeases function as antiport transporters, modulating transport efficiency^13^.

Melanin and phospholipase activity could not be tested because the wild type, complemented, and *cys3Δ* strains did not grow in the various media supplemented with cysteine and methionine. Urease activity and capsule synthesis were evaluated, and no difference was observed relative to the wild type.

Experiments in *G*. *mellonella* with wild type, mutant, and complemented strains, showed that *cys3Δ* mutant has virulence attenuated in invertebrate animal model (Fig. 4b). In summary, we concluded that the sulfate uptake plays an important role during virulence in *C*. *neoformans*, which is in accordance to previous data reported in the literature^23^.

### The cellular localization and abundance of GFP-Cys3 protein varies according to nutritional status

**Figure 5:**
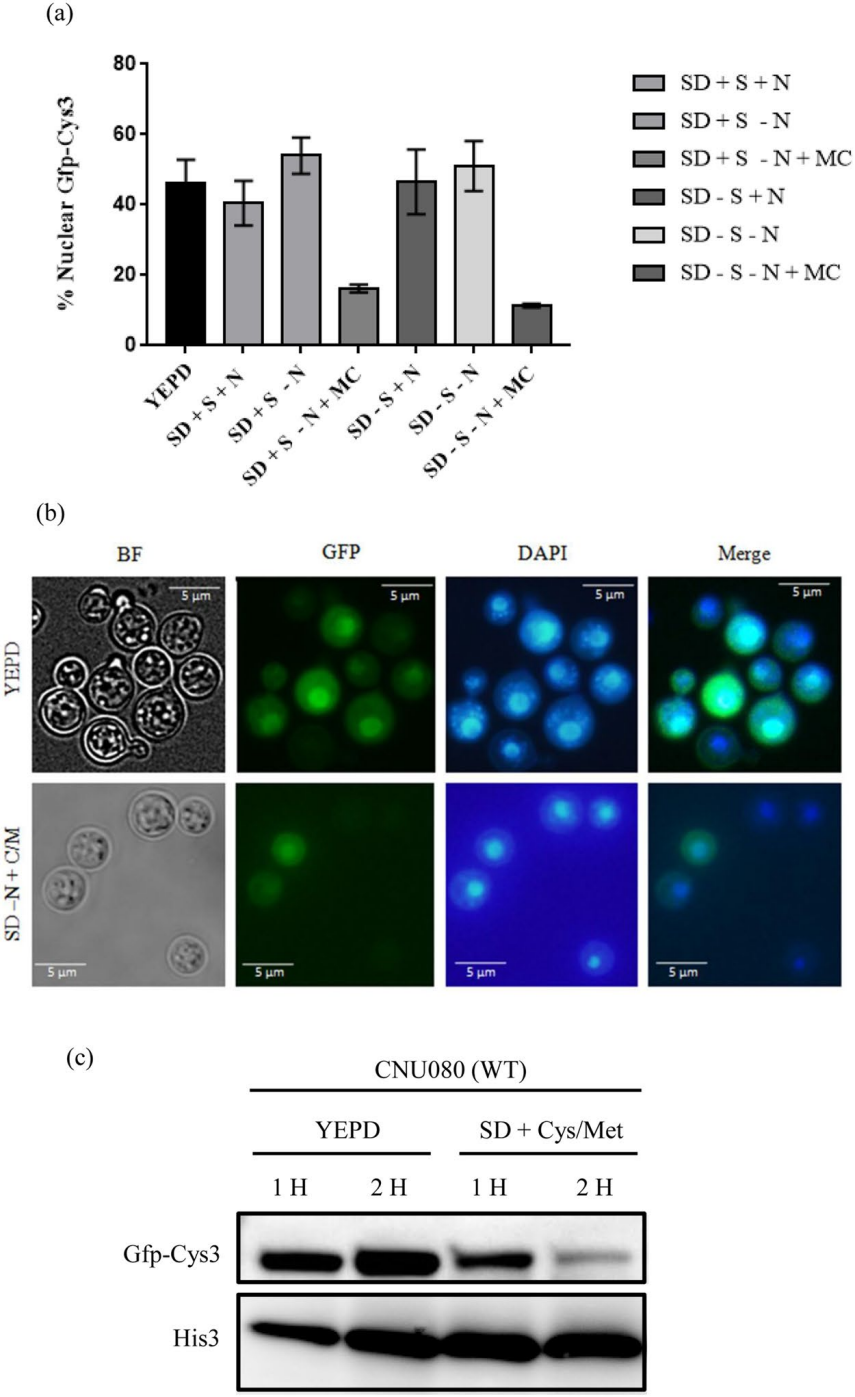
GFP-Cys3 cellular localization and expression pattern in western blotting. (**a**) Percentage of GFP and DAPI localization accessed by fluorescence microscopy in CNU080 submitted to various nutritional conditions (n > 100 cell/treatment, asterisks represent the significant differences (*p < 0.01 and ***p < 0.001). (**b**) Fluorescence microscopy images showing localization of GFP and DAPI in YEPD (top) and SD-N + Cys/Met (Bottom). Merge is the overlay of DAPI and GFP images and localization is represented by the yellowish color; (**c**) western blotting of proteins extracted from strain CNU080 cultivated for 1 and 2 h in YEPD and SD-N + Cys/Met. Mouse Anti-GFP primary antibody was used at 1:2000 dilution and secondary anti-mouse horseradish peroxidase-linked antibody was applied at 1:2000 dilution. Loading control was done by detecting histone H3 protein with rabbit anti-His3 antibody (1:2000) as primary and anti-rabbit secondary antibody horseradish peroxidase linked (1:2000). The panel is a crop of a bigger image. The original images are shown in Supplementary data.

To gain information about the sub cellular localization of Cys3 in response to nutritional status, a translational fusion protein of Cys3 with GFP at the N terminus was constructed (CNU080). In YEPD, GFP-Cys3 and DAPI localizations are coincident in 46% of the cells analyzed (Fig. 5a). Figure 5b illustrates the cells in bright field, GFP fluorescence, DAPI nuclear staining, and merged of the last two images.

We followed GFP-Cys3 by fluorescence microscopy in cells cultivated in regular (sulfate containing) and sulfate free SD medium with ammonium sulfate and ammonium chloride, respectively (SD + S + N vs SD-S + N); also GFP-Cys3 was evaluated in regular and sulfate free SD lacking the preferred nitrogen source (SD + S-N vs. SD-S-N). No statistical difference was detected in the percentages of nuclear GFP-Cys3 in cells growing in regular and sulfate free SD with preferred nitrogen source (40% vs. 46%) and for regular and sulfate free SD without the preferred nitrogen source (54% vs. 51%), as shown in Fig. 5a. These percentages are not statistically different from those obtained in complex medium (46%). These results suggest that sulfate itself does not influence GFP-Cys3 localization. However, the supplementation of SD + S-N and SD-S-N medium with sulfur amino acids as sole nitrogen source (+M/C) was enough to cause a statistically significant drop in the percentage of nuclear GFP-Cys3 (16% and 11% respectively, Fig. 5a); also, the overall fluorescence seems to be lost in the presence of sulfur amino acids (Fig. 5b). These data indicate that GFP-Cys3 localizes to the nucleus in complex medium (sulfur limiting), and it is probably active in this condition, inducing the sulfur uptake. However, in the presence of sulfur amino acids as sole nitrogen source, GFP-Cys3 is no longer localized in the nucleus since fluorescence was lost; likely the protein was degraded, shutting down the sulfur assimilation network.

Western blotting showed a 58 KDa band corresponding to the expected size of the GFP-Cys3 fusion. In complex medium, the amount of GFP-Cys3 increased between 1 and 2 hours post induction. However, in SD-N + Cys/Met, GFP-Cys3 band sharply decreased, especially after 2 hours post induction. These data are consistent with the fluorescence microscopy, since in complex medium GFP-Cys3 fluorescence not only coincided with the DAPI nuclear staining in higher percentages, but it is brighter than in SD-N + Cys/Met. The supplementation of methionine and cysteine to the medium targeted this protein for degradation, which is probably the reason why fluorescence signal was lost (Fig. 5c). Based on these results, YEPD and SD-N + M/C were used as the sulfur limiting and non-limiting condition, respectively, in further experiments.

### Proteomic analysis of the Cys3 protein complex

The data generated so far suggest that *C*. *neoformans* Cys3 must be highly regulated at the post-translational level. For more insights into this regulation and to identify putative regulatory partners, we used CNU080 (GFP-Cys3) cell extracts to immunoprecipitate (IP) a putative protein complex. IP was carried out with protein extracts collected in 2 different nutritional conditions. The proteins found in the immune complex were identified by LC/MS and MALDI/TOF and are depicted in Table 1. Several proteins were identified, but the current study explored and confirmed the interactions found among Cys3, the phosphatases Gpp2, and the calcineurin (Cna1 and Cnb1).

**Table 1:** Proteins identified by LC/MS and MALDI/TOF after IP of GFP-Cys3. CNU080 cell extracts were collected in two growth conditions (2 h in YEPD and SD).

Immunoprecipitation*	Amino acid sequence identified	% coverage
YEPD	Sulfate adenylytransferase (MET3)	14.80
	Elongation fator 1-alpha (TEF1)	32.13
	Glyceraldehyde-3-phosphate dehydrogenase (GPD)	22.12
	Cytochrome c peroxidase_mitochondrial (CCP1)	33.48
	40 S ribossomal protein S0 (RPS0)	31.33
	40 S ribossomal protein S1 (RPS1)	32.24
	ATP-dependent RNA helicase eIF4A (TIF1)	13.10
	ATP-dependent RNA helicase SUB2 (SUB2)	11.90
	Mitochondrial import inner membrane translocase subunit (TM8)	26.14
SD	Protein Phosphatase (*GPP2*)	11.44
	Serine/threonine protein phosphatase 2B catalytic subunit A1 (*CNA1*)	9.23
	Calcineurin subunit B1 (*CNB1*)	10.10

We used the Yeast Two-Hybrid assay to confirm the interactions identified. *C*. *neoformans CYS3* coding sequence was cloned into the bait plasmid (pRCP063), which allows the protein of interest to be expressed as a fusion with the Gal4 DNA binding domain in Y2HGold strain. Transformants were tested for toxicity (SDO) and auto activation (SDO/X/A) of the fusion protein. On SDO plates, the transformants grew to the same rate as the control (pGBKT7-53) after 3 days, demonstrating that the fusion protein DB-Cys3 is not toxic to *S*. *cerevisiae*. However, the auto activation assay yielded a large number of blue colonies on SDO/X/A plates, indicating that the bait plasmid is able to activate the reporter genes (*AUR1-C* and *MEL1*) by itself. On the other hand, no colonies grew on the SDO/X/A plates transformed with the positive control bait plasmid pGBKT7-53, validating the experiment. This result clearly indicates that Cys3 has an activation domain and functions as a potent transcriptional activator, which in proximity to the Gal4 DNA binding domain, promotes transcription of the reporter genes in the absence of the prey; therefore, it cannot be used in the two hybrid assay as bait. Despite that, this result states that *CYS3* gene encodes a protein with very strong activation capacity.

We cloned the *CYS3* cDNA into the pGADT7 (prey plasmid). Since this type of fusion protein lacks a Gal4 DNA binding domain, our hypothesis was that Cys3 auto activation would be abolished. Indeed, Cys3 did not auto activate the reporter system, enabling its use as AD-Cys3 fusion protein in a two hybrid assay. Gpp2, Cna1, and Cnb1 were expressed as DB and AD fusion proteins by cloning their coding sequences in pGBKT7 and pGADT7. Cna1 has an auto inhibitory domain at the C terminus, which blocks its interaction with the regulatory subunit Cnb1. An earlier report in the literature mapped this domain and found that its elimination allows Cna1 interaction with Cnb1 in the absence of calcium^69^. Therefore, in addition to a full-length *CNA1*, we also constructed a truncated version of this gene lacking 666 nucleotides at the 3´end of the coding sequence. All constructs were tested for auto activation and toxicity in *S*. *cerevisiae*, and none were toxic or auto activated *AUR1-C* or *MEL1* reporter genes, demonstrating that they are all useful for two hybrid system interactions. Pairs of plasmids expressing fusion proteins were co-transformed into Y2HGold, tested for all four reporters (*AUR1-C*, *MEL1*, *ADE2*, and *HIS3*), and the results are presented in Table 2.

**Table 2:** Protein interactions, plasmids used, and the results obtained in this work.

Interacting proteins	Plasmids pGBKT7/pGADT7	Autoactivation/Toxicity	Interaction*
Cnb1 & Cna1	pRCP089/pRCP065	No/No	−
Cna1-ΔC & Cnb1	pRCP092/pRCP096	No/No	+
Cna1 & Gpp2	pRCP073/pRCP088	No/No	−
Cna1-ΔC & Gpp2	pRCP092/pRCP088	No/No	+
Cnb1 & Gpp2	pRCP089/pRCP088	No/No	+
Cna1 & Cys3	pRCP073/pRCP076	No/No	−
Cna1-ΔC & Cys3	pRCP092/pRCP076	No/No	−
Cnb1 & Cys3	pRCP089/pRCP076	No/No	+
Gpp2 & Cys3	pRCP071/pRCP076	No/No	+

*- = negative interactions and + = positive interactions.

**Figure 6:**
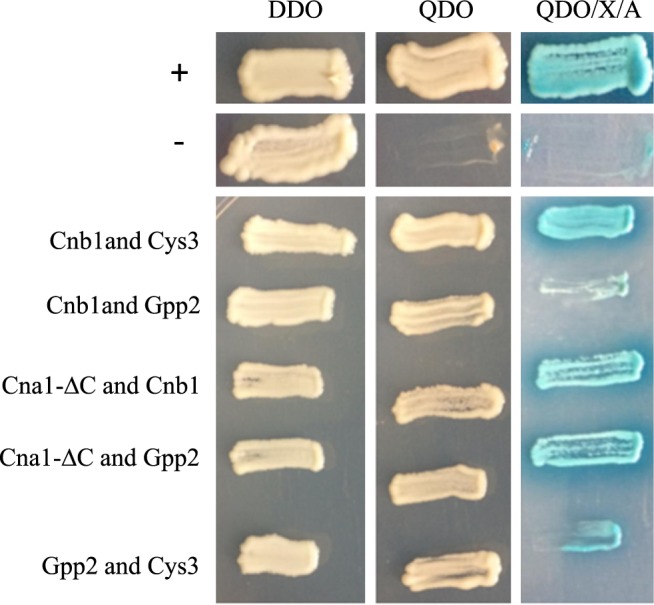
Yeast two-hybrid assay. Images depict representative colonies of *S*. *cerevisiae* expressing pairs of bait and prey fusion proteins, which are described at the left side of the panel. Each column represents a plate composition; DDO = double drop out (minus tryptophan and leucine), where no reporter gene is activated; QDO = quadruple drop out (minus tryptophan, leucine, histidine, and adenine), where two reporters are activated; and QDO/X/A = quadruple drop out with aurobasidin and X-α-Gal, where all four reporters are active. + represents the positive and − the negative control provided by Match Maker kit (Clontech).

Figure 6 shows a representative transformant of each positive interaction on DDO, QDO, and QDO/X/A plates. On DDO, no reporter genes were tested, and all colonies grew, including the positive and negative controls. On QDO plate, two reporter genes were evaluated (*HIS3* and *ADE2*), and QDO/X/A represents the positive activity of 4 reporters (*HIS3*, *ADE2*, *AUR1-C*, and *MEL1*). In the last two conditions, all transformants grew, except the negative control. Therefore, positive (pGBKT7-53 and pGADT7-T), the negative control (pGBKT7-Lam and pGADT7-T), and the interaction of Cna1 and Cnb1 (pRCP092 and pRCP096) behaved as expected. Positive interactions were found between Cna1 and Gpp2; Gpp2 and Cnb1; Cnb1 and Cys3; and Gpp2 and Cys3. These data confirm that Cys3 physically interacts with the regulatory subunit of Calcineurin and the Gpp2 phosphatase, confirming the results found by mass spectrometry.

### Calcineurin and Gpp2 modulate the cellular localization and the abundance of GFP-Cys3

Assuming that Cys3 interacts with calcineurin and Gpp2 and a regulatory relationship exists among these proteins, gene deletions of calcineurin and *GPP2* may cause differences in GFP-Cys3 localization and protein profile in western blots. Therefore, the catalytic and regulatory subunits of calcineurin (*CNA1* and *CNB1* respectively) and *GPP2* genes were individually deleted from CNU080 strain (GFP-Cys3). Strain CNU119 and CNU121 (*cna1Δ*::*Nat*^R^ and *cnb1Δ*::*Hph*^R^, respectively) presented growth arrest at 37 °C, as expected^67^.

**Figure 7:**
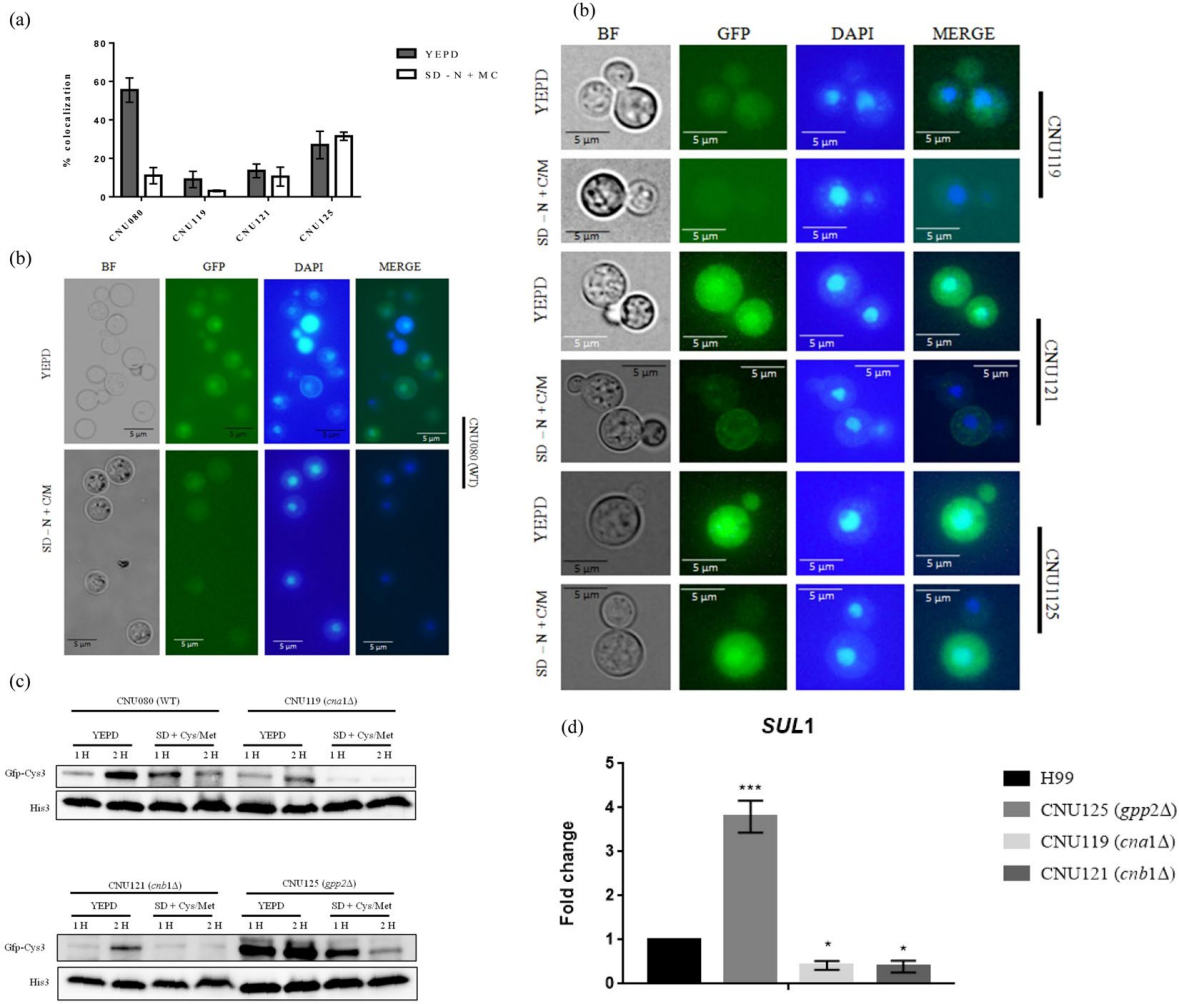
GFP-Cys3 cellular localization and expression pattern in western blotting in mutant background. (**a**) Bars represent percentage of GFP and DAPI localization accessed by fluorescence microscopy in CNU080 (wild type), *cna1Δ* (CNU119), *cnb1Δ* (CNU121), and *gpp2Δ* (CNU125) cultivated for 2 h in YEPD and SD-N + Cys/Met; (**b**) fluorescence microscopy images showing localization of DAPI and GFP in YEPD (left) and SD-N + Cys/Met (right side). Merge is the overlay of DAPI and GFP images and localization is represented by the yellowish color; (**c**) western blotting of total proteins extracted from strain CNU080, CNU119, CNU121, and CNU125 cultivated for 1 and 2 hours in YEPD and SD-N + Cys/Met. Mouse Anti-GFP primary antibody was used at 1:2000 dilution and secondary anti-mouse horseradish peroxidase-linked antibody was applied at 1:2000 dilution. Loading control was done with histone H3 protein with rabbit anti-His3 antibody (1:2000) as primary and anti-rabbit secondary antibody horseradish peroxidase linked (1:2000). The original western blot images are shown in Supplementary data; (**d**) expression pattern by qPCR of *C*. *neoformans* sulfate permease (*SUL1*) in wild type (H99), *gpp2Δ* (CNU125), *cna1Δ* (CNU119), and *cnb1Δ* (CNU121) in YEPD. Asterisks denote the statistically significant differences encountered between wild type H99 and the mutants. *p < 0.05, ***p < 0.001.

Fluorescence microscopy showed that after 2 hours of incubation in YEPD, around 55% of wild type (CNU080) cells presented GFP-Cys3 in the nucleus (Fig. 7a,b), whereas, *cna1Δ* (CNU119) and *cnb1Δ* (CNU121) had 9% and 13% of nuclear GFP-Cys-3, respectively, which are statistically significant differences relative to wild type (*p* < 0.001). Deletion of *GPP2* (*gpp2Δ* CNU125) also caused a significant reduction (26%) in DAPI and GFP-Cys3 coincidence, relative to wild type (55%); however, this reduction was not as sharp as that found for calcineurin mutants (Fig. 7a,b).

In SD supplemented with sulfur amino acids (SD-N + Cys/Met), *cna1Δ* (CNU119) and *cnb1Δ* (CNU121) had 3% and 10%, respectively, of nuclear GFP-Cys3 compared to wild type (11%); these differences were not statistically significant relative to wild type (p < 0.001). However, the GFP-Cys3 protein, in CNU125 strain (*gpp2Δ*) grown in SD-N + Cys/Met had an increase in nuclear GFP-Cys3 (31.5%) relative to wild type strain (11%), which was a statistically significant difference (Fig. 7a,b).

GFP-Cys3 was detected by western blotting in wild type, *cna1Δ*, *cnb1Δ*, and *gpp2Δ* (CNU080, CNU119, CNU121, and CNU125, respectively). In the wild type strain (CNU080) at 2 hours post incubation in YEPD, the intensity of GFP-Cys3 band increased (Figs 5c and 7c); on the other hand, GFP-Cys3 accumulation decreased in SD supplemented with cysteine and methionine (Figs 5c and 7c). In the *cna1Δ* mutant, GFP-Cys3 was dramatically reduced in YEPD and in SD + Cys/Met relative to wild type (Fig. 7c). Similar results occurred for *cnb1Δ* in YEPD and SD + Cys/Met (Fig. 7c). These results suggest that calcineurin complex is probably active and required to maintain Cys3 protein levels and nuclear localization in YEPD. Conversely, in the *gpp2Δ* mutant (CNU125), GFP-Cys3 band appeared to be more intense compared to wild type at 1 and 2 hours post incubation in YEPD. Whereas in the SD-N + Cys/Met medium, at 1 hour, the GFP-Cys3 band was more intense than wild type, with no striking changes at 2 hours in the same condition (Fig. 7c). This suggests that Gpp2 phosphatase is required during growth in YEPD and SD-N + Cys/Met to target Cys3 to degradation.

If the calcineurin complex is responsible for maintaining Cys3 in the nucleus at high levels in complex medium, where it actively induces gene expression, the deletion of *CNA1* and *CNB1* would negatively affect the expression of Cys3 targets, such as the sulfate permease *SUL1* in YEPD. Conversely, Gpp2 seems to be responsible for somehow inactivating Cys3 by targeting it to degradation; therefore, its deletion led to a positive effect over the expression of Cys3 targets. *SUL1* expression in *cna1Δ* and *cnb1Δ* was 0.41 and 0.39 fold reduced, respectively, which were expression levels significantly lower than wild type, but were not significantly different from the levels encountered in *cys3Δ* (Figs 7d and 3a), suggesting that *cna1Δ* and *cnb1Δ* deletions caused a similar effect to the *cys*3 deletion. On the other hand, *GPP2* deletion caused a 3.7 fold increase in *SUL1* expression, consistent with our hypothesis that Gpp2 is responsible for Cys3 inactivation (Fig. 7d).

### Transcriptional profile of *cys3Δ*

The global transcriptional profile of *cys*3Δ was characterized by RNAseq. Enrichment analysis, considering two-fold DEGs (Differentially Expressed Genes), showed 11 repressed and 36 induced genes in the mutant strain (Supplementary Table 4 and supplementary Table 5, respectively). Five of these 11 repressed genes, encode enzymes of the sulfur amino acid biosynthetic pathway: *MET2*, *MET10*, *MET3*, *STR1*, and *CYS3*, suggesting that Cys3 transcription factor controls gene expression in at least three branches of the sulfur uptake and sulfur amino acid biosynthesis (Fig. 1). However, if we consider less than two-fold change in gene expression, *SAM1* (S-adenosylmethionine synthase) rises as a down regulated gene (25% reduced relative to wild type *p* = 0,0005), suggesting that Cys3 also controls the methyl cycle. In addition, a sulfide quinone oxidoreductase was repressed, suggesting that Cys3 acts upon hydrogen sulfide metabolism generating thiosulfate, which could be used as sulfur source.

Among the DEGs that were induced, two of them are involved in aromatic amino acid biosynthesis (3-deoxy-7-phosphoheptulonate synthase, *ARO3*, and *ARO4* in *S*. *cerevisiae*); one is part of the lysine biosynthetic pathway (homocitrate synthase); aspartate kinase and asparagine synthetase are involved in aspartate and asparagine biosynthesis, respectively. The first one catalyzes the first committed step of the methionine and threonine common pathway (homoserine biosynthesis) and the second catalyzes the synthesis of L-asparagine from L-aspartate. In addition, 18 up regulated DEGs encode hypothetical proteins. Of special interest among the induced genes are (i) a taurine catabolism dioxygenase, which is encoded by *JLP1* in *S*. *cerevisiae*, involved in sulfonate catabolism for use as a sulfur source^70^; (ii) a sulfiredoxin, which contributes to oxidative stress resistance by reducing cysteine-sulfonic acid groups^71^; and a (iii) transcriptional repressor denominated *CLR6* known to control virulence and various amino acid biosynthetic routes in *C*. *neoformans*^72^.

A search for GO term (biological process and molecular function) and KEGG pathway database reflects the results described above and are shown in Tables 3 and 4.

**Table 3:** List of the biological processes and molecular functions found to be differentially expressed (up and down regulated), according to Gene ontology (GO).

GO ID	GO Term Biological Process	Genes Found
**Upregulated**		
GO:0008652	cellular amino acid biosynthetic process	5
GO:0046394	carboxylic acid biosynthetic process	5
GO:0016053	organic acid biosynthetic process	5
GO:0044283	small molecule biosynthetic process	5
GO:0006520	cellular amino acid metabolic process	5
GO:0009067	aspartate family amino acid biosynthetic process	2
GO:0009066	aspartate family amino acid metabolic process	2
GO:0019752	carboxylic acid metabolic process	5
GO:0043436	oxoacid metabolic process	5
GO:0006082	organic acid metabolic process	5
GO:0009073	aromatic amino acid family biosynthetic process	2
GO:0019878	lysine biosynthetic process via aminoadipic acid	1
**Downregulated**		
GO:0000103	sulfate assimilation	5
GO ID	GO Term Molecular Function	Genes Found
GO:0003849	3-deoxy-7-phosphoheptulonate synthase activity	2
GO:0016765	transferase activity, transferring alkyl or aryl (other than methyl) groups	2
GO:0004072	aspartate kinase activity	1
GO:0032542	sulfiredoxin activity	1
GO:0004410	homocitrate synthase activity	1
GO:0004066	asparagine synthase (glutamine-hydrolyzing) activity	1

**Table 4:** List of the KEGG pathways found to be differentially expressed according to the RNAseq analysis.

KEGG ID	Pathways Names	Size	Genes	FDR
**Upregulated**				
10	Glycolysis/Gluconeogenesis	51	1	0.0574747
250	Alanine, aspartate, and glutamate metabolism	30	1	0.0304203
300	Lysine biosynthesis	11	1	0.0117582
400	Phenylalanine, tyrosine, and tryptophan biosynthesis	17	1	0.0141679
620	Pyruvate metabolism	33	1	0.0293563
4113	Meiosis - yeast	127	1	0.210681
**Downregulated**				
920	Sulfur metabolism	15	1	0.0141679
230	Purine metabolism	93	1	0.143853
260	Glycine, serine, and threonine metabolism	24	2	0.00525596
270	Cysteine and methionine metabolism	31	1	0.0293563

## Discussion

Nutritional adaptation plays a crucial role in survival of microorganisms that are prone to occupy multiple ecological niches, since availability of nutrients may vary widely in these different environments. Metabolic plasticity, such as the ability to engage the biosynthesis, uptake or recycling of multiple nutrients confers advantage to the organisms living in diverse conditions. In previous works, our group and others have shown that amino acid biosynthesis and uptake are both very important biological processes that permit *C*. *neoformans* to adapt to the animal host. The methionine biosynthetic pathway has been linked to the ability of this opportunist yeast to survive and cause disease; however, the regulatory aspects of sulfur uptake and sulfur amino acid biosynthesis were unknown.

Rich medium is probably the most sulfur limiting condition for *C*. *neoformans*, since *CYS3* gene was required in YEPD, and *CYS3* and *SUL1* expression were consistently induced in this nutritional condition (Figs 2a and 3). Moreover, western blots and fluorescent microscopy demonstrated that GFP-Cys3 protein localizes to the nucleus in rich medium, probably acting as a transcriptional activator of the sulfur uptake pathway. In SD, either in the presence or absence of inorganic sulfate, GFP-Cys3 remains in the nucleus at high percentages. GFP-Cys3 only lost its nuclear localization and was degraded when sulfur amino acids as sole nitrogen source were added to the medium, as shown in Fig. 5. This set of data suggests that YEPD medium has inorganic sulfate sources and has low amounts of sulfur amino acids, triggering sulfur uptake and *de novo* biosynthesis of methionine and cysteine. In this nutritional condition, amino acid permeases are repressed, likely, making a small contribution to sulfur amino acid transport^15^. However, when sulfur amino acids are present in a condition that favors its transport (sole nitrogen source and NCR relief), GFP-Cys3 is inactivated probably by protein degradation. Therefore, this set of data suggests that sulfur amino acids may be the primary sulfur source in *C*. *neoformans*, which triggers inhibition of sulfur assimilation pathway controlled by Cys3; reduced pools of methionine and cysteine lead to sulfate uptake and sulfur amino acid biosynthesis also controlled by Cys3 transcription factor. In *N*. *crassa*, sulfur limitation results in activation of *cys-3*, expression of its targets, such as sulfate transporter *cys-14*, and autogenous regulation of c*ys-3*. Supplementation of high levels of inorganic sulfate and methionine trigger *cys-3* degradation^29,30,32,73^. The same is true for *S*. *cerevisiae MET4* (*N*. *crassa cys-3* homologue), which is ubiquitinated by *MET30* and targeted to degradation in the presence of S-adenosylmethionine, methionine, and cysteine^27,55^. Taken together these data indicate that, while *C*. *neoformans*, *S*. *cerevisiae*, and *N*. *crassa* have different genetic elements controlling sulfur uptake, similar nutritional signals cause Cys3 activation and inactivation.

In addition, growth curves and sulfur amino acid supplementation experiments allowed us to conclude that cysteine is a better sulfur source than methionine, and so is homocysteine, which complements growth to the wild type levels. These results imply that the transulfurylation pathway operates efficiently in *C*. *neoformans*. However, the fact that methionine only partially rescued growth in a *cys3Δ* in the presence of non preferred carbon and nitrogen source at higher temperature (Fig. 2a) suggests that part of the low growth rate is due to low methionine transport. However, even when transport is increased, growth is still low. The inefficient methyl cycle function may be another explanation for this observation. The *met3Δ* mutant, which is also unable to generate sulfide, grows better than *cys3Δ* in methionine, likely due to differences between these two mutants^22^. A search for DEGs that have less than two-fold change compared to wild type found that *SAM1* (S-adenosylmethionine synthase) is 25% down regulated in the *cys3Δ* mutant, suggesting that Cys3, at least partially, controls the methyl cycle. Toh and collaborators reported that methionine is a poor sulfur source for *cys1Δ* mutant (*MET17*), which is in accordance to our observation^40^. Probably, low growth rate of *cys1Δ* is also due to low amino acid transport. For the transulfurylation pathway, our data indicate that it operates efficiently, as homocysteine and cysteine fully restored growth.

Previous works showed that mutations that block methionine and cysteine biosynthesis render strains unable to express virulence factors and are avirulent in animal model^21–23^. With this in mind, we evaluated response of the *cys3Δ* strain to various types of stress that pathogens encounter in the host environment, such as oxidative, osmotic, saline, and alkaline stresses, and, except for pH stress, none of them seem to differentially affect the mutant strain relative to wild type. Since cysteine is a very important substrate for glutathione pathway, one would think that oxidative stress response would be compromised in the mutant; however, there was no difference between mutant and wild type strains. Probably, supplementation of 20 mM cysteine to the growth medium is sufficient to provide enough cysteine to the glutathione pathway and also to produce methionine by the transulfurylation pathway. In the plant pathogen *A*. *alternate*, methionine and cysteine deficiency led to increased sensitivity to H_2_O_2_, suggesting that these auxotrophs affect oxidative stress response^59^.

Invertebrate animal model demonstrated that *cys3Δ* is hypovirulent, which is consistent with other mutants that block sulfur amino acid biosynthesis that are avirulent in animal model^22,23^. In *A*. *fumigatus* deletion of *metR* (*CYS3* homologue) is essential for pulmonary aspergillosis dissemination, similar to our data in *G*. *mellonella* and previous work where a functional sulfur amino acid biosynthesis is important for virulence^22,23,59,74^. Likely, the inability to synthesize sulfur amino acids plays multiples roles on *C*. *neoformans* adaption and survival in the host, from not having enough sulfur amino acids for proteins synthesis to difficulties in combating oxidative stress through glutathione pathway, lipid and polyamine biosynthesis and methylation processes, which are all crucial to cell survival.

We employed mass spectrometry and yeast two hybrid assay to identify and confirm Cys3 protein interactions to hopefully shed some light on the genetic elements that regulate sulfur amino acid biosynthesis. The lack of information on amino acid biosynthesis regulation and its importance for fungal pathogenesis justify the efforts put into this task^21–23,59,74^. To our surprise, the proteomics approach identified the widely known calcineurin complex^67,75^ and the protein phosphatase Gpp2 (glycerol-3-phosphatase) that had not been described in *C*. *neoformans* previously. In *S*. *cerevisiae*, Gpp2 is involved in osmotic, oxidative, and diauxic stress^65^. Park and collaborators used phosphoproteomics analysis to identify the calcineurin targets, and Gpp2 was considered one of them, since it was hyper phosphorylated under Cna1 inhibition, suggesting that Gpp2 is dephosphorylated by the calcineurin complex^76^. Our data show that both, catalytic and regulatory calcineurin subunits are necessary to maintain proper Cys3 protein levels in rich medium, a condition that we believe is sulfur limited and Cys3 activity is essential. Calcineurin is also required to keep Cys3 in the nucleus in high levels, since *cna1Δ* and *cnb1Δ* mutants have very low percentage of nuclear GFP-Cys3 in YEPD, according to our fluorescence microscopy, and very low protein abundance relative to wild type, in western blot. Although, we were surprised to find the calcineurin in association with Cys3, this signaling phosphatase has been linked to transcriptional activation before. Crz1 is a transcriptional activator that has to be dephosphorylated by calcineurin to enter the nucleus and control gene expression^77^.

**Figure 8:**
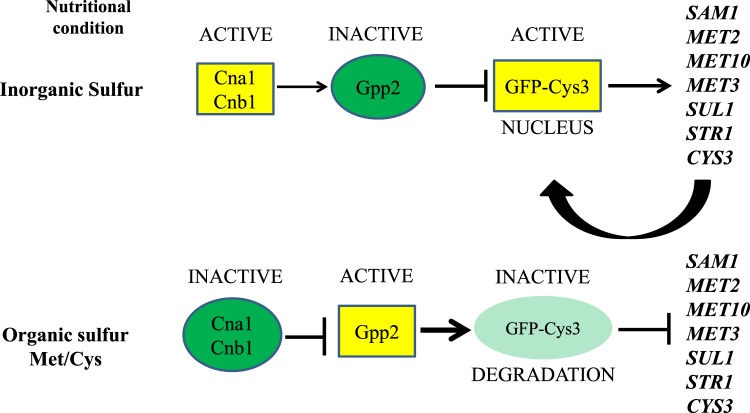
Model of the sulfur amino acids biosynthesis regulation in *C*. *neoformans*.

Taken together, the data generated by proteomics, mutant phenotyping, fluorescence microscopy, and western blots allowed us to assemble a model for future experimentation (Fig. 8): in rich medium, calcineurin would be responsible to maintain Gpp2 inactive and unable to, somehow, affect Cys3, which would accumulate in a certain form that favors its nuclear localization and high protein levels. On the other hand, in the presence of sulfur amino acids, calcineurin would be inactive and unable to dephosphorylate Gpp2; it would somehow, affect Cys3 function, which would be targeted to degradation. Along with this rational, *SUL1* (the target of Cys3) would be down regulated in the calcineurin mutants (*cna1Δ* and *cnb1Δ*) and up regulated in *gpp2Δ*, which is exactly what we observed (Fig. 7d), reinforcing our hypothesis (Fig. 8).

In this paper, we showed that *CYS3* encodes a transcriptional activator that, (i) once deleted, causes a double auxotroph (methionine and cysteine), rendering strains unable to grow in complex medium. (ii) Cys3 is the major regulator of inorganic sulfur uptake, and the best organic sulfur sources for *C*. *neoformans* are cysteine and homocysteine. According to our analysis, (iii) all predicted branches of sulfur amino acid biosynthetic pathway (Fig. 1) are functional, allowing cysteine and methionine synthesis from serine and aspartate, as long as sulfide is generated by the sulfur assimilatory pathway controlled by Cys3. The lack of Cys3 compromises the sulfur amino acid biosynthesis; (iv) transcriptional profiling showed that deletion of Cys3 leads to failure in activating key genes in sulfur amino acid biosynthesis and induction of various amino acid biosynthetic routes, an effect similar to GAAC (Global Amino Acid Control) described in *S*. *cerevisiae*, in which the low levels of one or more amino acids leads to induction of several biosynthetic pathways^78,79^. (v) Cys3 has a complex post translational regulation, involving protein localization and degradation dictated by calcineurin and Gpp2 phosphatases, which responds to sulfur amino acids. (vi) in addition to protein synthesis and structure, the lack of sulfur amino acids has the potential to affect the oxidative stress response (glutathione pathway), polyamine biosynthesis, and all the methylations occurring in the cell (S-adenosylmethionine synthesis). These are important biological processes for survival in the host; therefore, we believe that avirulence of the sulfur auxotroph strains in animal model observed in this work and others is ultimately due to their inability to cope with the multiple stresses, including nutritional^22,23,40,58–61^. These finding underline the importance of unraveling the details of nutritional signaling and its connection with virulence in *C*. *neoformans*.

## Methods

### Strains, medium composition, and growth conditions

The strains, plasmids, and primers are listed in Supplementary Tables 1–3 respectively. Routine growth was carried out on YEPD (1% yeast extract, 2% bacto-peptone, and 2% glucose). Synthetic dextrose (SD) was prepared with yeast nitrogen base, YNB (0.67 g/L yeast nitrogen base with or w/o amino acid and ammonium sulfate, depending on experimental design, 2% glucose and 10 mM of each amino acid as sole nitrogen source) unless specified otherwise, at 30 °C or 37 °C on plates or on liquid medium with 150 rpm in a rotary shaker. YEPG was prepared with 2% of galactose instead of dextrose. Sulfur amino acids were supplemented at 20 mM each and homocysteine at 25 mM. Sulfate free medium was made by substituting all sulfate reagents for chloride in the stock solutions.

### Genetic manipulation

Gene disruption cassettes were generated by double joint PCR as previously described^80^. Deletion constructs were introduced into wild type H99 or CNU080 (GFP-Cys3) by biolistic transformation^81^, transformants were selected on YEPD plates supplemented with G418, Hygromycin, or Nourseothricin (200 µg/mL, 100 µg/mL, or 200 µg/mL, respectively), except for *cys3Δ*::*Neo*^R^ mutants that were selected on SD with G418, sulfur amino acid, serine, and proline as sole nitrogen source (10 mM). Complemented strains were obtained by transforming the CNU123 strain with a PCR amplified wild type allele of *CYS3* and a co-transforming plasmid (pZPHyg). Selection was done in YEPD with 200 µg/mL of Hygromycin B. Confirmation was done by colony PCR and real time qPCR with primers PRCP309 and PRCP310.

GFP-Cys3 translational fusion was done by PCR amplifying *CYS3* wild type allele from H99 gDNA and CloneAmp, a proofreading enzyme (Takara). This amplification represents a GFP fusion at the 5´ end of the *CYS3* gene; it includes the start and stop codons plus a 300 bp of the terminator region. The 1289 bp fragment was cloned into pCN50 digested with *Bam*HI using InFusion cloning kit (Takara). The resulting plasmid was introduced in H99 and transformants were submitted to fluorescent microscopy to select the green fluorescent ones. CNU080 was chosen for further analysis.

### *In vitro* and *in vivo* virulence tests

All assays involving *cys3Δ*::*Neo*^R^ strains (CNU123 and CNU124) were supplemented with 20 mM cysteine. Thermotolerance was evaluated in rich medium (YEPD) and SD + SAA (Sulfur amino acids) at temperatures of 30 °C and 37 °C. Urease activity was assayed in urea agar base^82^. Induction of capsule was carried out at 37 °C in a shaker (150 rpm) in Sabouraud Broth, diluted in MOPS (1:10)^83^. Cell samples were collected at 24 hours, stained with India ink, and analyzed by light microscopy (Olympus BX51M). All measurements were performed in biological triplicates with the assistance of CellSens software (Olympus). The data were treated statistically using ANOVA (GraphPad Prism 7).

Multi-stress sensitivity was evaluated with YEPD or SD medium (plus 20 mM cysteine) supplemented with 0.75 M and 1.5 M of NaCl or KCl. The cell wall sensitivity was evaluated on YEPD plus 0.5% Congo Red. All plates were incubated at 30 °C and 37 °C.

*In vivo* assays with *G*. *mellonella* were conducted according to previous published protocol^84^. Briefly, mutant, wild type, and complemented strains were inoculated into 5 mL of SD plus 20 mM cysteine and incubated with orbital agitation 150 rpm for 16–18 h. Subsequently, suspensions were collected by centrifugation, washed twice in sterile PBS and adjusted to 1 × 10^6^ cell/mL in PBS supplemented with ampicillin (20 mg/kg body weight). Groups of 16 caterpillars with 200 mg of average weight were inoculated with 10 μL of the suspension with the aid of a Hamilton syringe in the region of the last pro-paw. Thereafter, caterpillars were separated on glass Petri dishes (15 mm diameter) and incubated at 30 °C and 37 °C for 8 days. They were monitored daily by observing spontaneous or induced movements with a previously sterilized clip. The experiment was completed when the larvae died or formed cocoons.

### Immunoprecipitation and mass spectrometry (LC/MS and MALDI-TOF)

WT (H99) and CNU080 cell were grown in YEPD at 30 °C overnight, the cells were harvested, washed twice in PBS, and resuspended in the appropriate induction medium (YEPD or SD). After a 2 h period, 10 mL of culture was harvested and resuspended in 500 μl of lysis buffer (50 mM Tris-Cl pH 7.4, 100 mM NaCL, 0.5 mM PMSF, 0.5% NP 40, 10 mM Orthovanadate, 50 mM NaF, 1x Protease inhibitor) and glass beads (0.5 mm). Cell suspension and beads were beat for 1 minute, alternating with 2 minutes on ice (Mini-bead beater - BioSpec) for 10 cycles. Cell extracts were centrifuged for 2 minutes (13000 rpm), the supernatant was recovered and protein concentration was determined by Bradford assay (BioRad).

SDS-PAGE was performed to evaluate the protein integrity and also to analyze the results of immunoprecipitation. In brief: 500 µg of protein extracts (H99 and CNU080) were incubated with 10 µL of GFP-Trap®_A (Chromotek) according to the instructions provided by the manufacturer. Laemmle sample buffer (v/v) was added to immunoprecipitated reaction and the mixture was heated for 5 minutes at 95 °C and separated on SDS-PAGE (12%) in running buffer (25 mM Tris, 192 mM Glycine, 0.1% SDS) at 80 V for 2 h. After gel staining with Coomassie blue, the bands that appeared only in the CNU080 immunoprecipitated extract were excised from SDS-PAGE and subjected to reduction in 10 mM dithiothreitol for 30 min at 56 °C, alkylation in 50 mM iodoacetamide for 30 min at room temperature in the dark, and overnight in-gel digestion with trypsin (Promega), in 50 mM ammonium bicarbonate at 37 °C. Tryptic peptides were extracted with 5% formic acid/50% acetonitrile at room temperature for 45 min. The supernatant of the extraction was concentrated in a vacuum centrifuge and desalted using ZipTip C-18 tips (Millipore). The LC-MS^E^ experiments were performed on a Synapt G2 mass spectrometer coupled to a nanoAcquity UPLC system (Waters) as described previously^85^. Briefly, the peptide mixture was loaded online for 5 min at a flow rate of 8 μl/min of phase A (0.1% formic acid) using a Symmetry C18 trapping column (Waters). The trapped peptides were subsequently separated by elution with a gradient of 7 to 35% of phase B (0.1% formic acid in acetonitrile) through a BEH 130 C18 column (1.7 μm particles, 75 × 150 mm; Waters) in 12 min, at 275 nl/min. Data were acquired in the data-independent mode in the m/z range of 50 to 2000 in resolution mode. Collision energies were alternated between 4 eV and a ramp of 17 to 45 eV for precursor ion and fragment ions, respectively, using scan times of 1.25 s.

MS data were processed by ProteinLynx Global Server v3.0.3 using low energy threshold of 750 counts and elevated energy threshold of 50 counts. MS/MS data were submitted to database search against *C*. *neoformans* sequences in the UniProtKB/Swiss-Prot database (www.uniprot.org). The following search parameters were used: carbamidomethylation of cysteines as fixed modification, oxidation of methionine, and N-terminal acetylation as variable modifications; up to 2 missed cleavage sites allowed for trypsin digestion, automatic fragment, and peptide mass tolerances. A maximum false discovery identification rate was set to 1%, estimated by a simultaneous search against a reversed database.

Alternatively, immunoprecipitates were obtained, directly digested, and loaded into MALDI-TOF 96 well plates covered with α-Cyano-4-hydroxycinnamic acid matrix (1 µL:1 µL), where the peptides were ionized and their masses were identified in a MALDI-TOF Autoflex Speed (Bruker Daltonics). The peaks detected were identified by the Bruker Daltonics BioTools 3.2 MS program and compared with Swiss-Prot data. The score of the results was given by probability (the protein score is 10 log (p), where *p* is the probability that the observed similarity is a random event). Scores greater than 56 were consider significant (p < 0.05).

### Fluorescent microscopy

CNU080 strain (GFP-Cys3) was incubated in several nutritional conditions depending on experimental design. Cells were grown overnight in 50 mL YEPD at 30 °C with 150 rpm rotation, wash with PBS three times, diluted to OD_600nm_ = 1 (50 mL), and incubated in various nutritional conditions at 30 °C for 1 and 2 h. A 100 µL aliquot was withdrawn for microscopy analysis. The cells were fixed with 4% formaldehyde (Sigma) (v/v) for 10 min at room temperature, washed twice with PBS and stained with 50 ηg/µL of DAPI (4′,6-Diamidine-2′-phenylindole dihydrochloride) for 10 min at room temperature. Cells were washed to remove the excess dye, and slides were prepared with 4 µL of ProLong antifade and 6 µL of the processed sample. Cells were viewed by direct fluorescent microscopy using an Olympus BX51M microscope and analysis was performed using Olympus CellSens, PhotoShop CS6, and ImageJ software. Percentages of nuclear GFP-Cys3 were obtained by counting the cells in which GFP and DAPI were overlaid (n > 100 cells/replicate). All microscopy experiments were done in triplicates.

### Western blot assay

Protein extract were obtained and separated on SDS-PAGE as described in the previous section. The gels were equilibrated in transfer buffer (48 mM Tris, 39 mM glycine, 20% methanol) and proteins were transferred to nitrocellulose membranes on Trans-Blot® SD Semi-Dry Electrophoretic Transfer Cell (BioRad) 15 V for 1 h. The membrane was blocked with 5% non-fat dry milk in TBS (10 mM Tris, 150 mM NaCl, pH 7.4) for 1 h at room temperature. The primary antibody (mouse anti-GFP ThermoFisher, 1:2000 dilution) was incubated overnight at 4 °C in 1% BSA. After three washes, 5 min each, in TBST (TBS with 0.1% Tween 20), the secondary antibody (goat anti-mouse-HRP, Cell Signaling Technology 1:2000 dilution) was incubated in TBST with 5% non-fat dry milk for 1 h at room temperature followed by four 5 min washes in TBST. Detection of chemiluminescence was performed by SuperSignal West Pico PLUS Substrate (ThermoFisher) using UviTec Systems. Loading control was done with mouse anti-Histone H3 antibody (1:2000) and rabbit anti mouse secondary antibody HRP –linked (1:2000).

### Yeast two hybrid assay

According to manufacturer’s instructions (ThermoFisher), pGBKT7 (bait) and pGADT7 (prey) vectors were digested with *BamHI* and *EcoRI*. Linear plasmids were cleaned with QiaQuick (Quiagen). *CYS3* (863 bp), *CNA1* (1920 bp), *CNA1ΔC* (1254 bp), *CNB1* (528 bp), and *GPP2* (1236 bp) cDNAs were amplified by RT-PCR (SuperScript II reverse transcriptase kit, ThermoFisher) from total RNA extracted from H99. Bands of the correct size were excised from agarose gel, purified with QIAquick Gel extraction kit (Qiagen), cloned into linear pGBKT7 or pGADT7 with In-fusion kit (Takara Clontech), and introduced in *E*. *coli* DH5α strains.

Plasmids were introduced in *S*. *cerevisiae* Y2HGold, which contains the 4 reporter genes (*ADE2*, *HIS3*, *MEL1*, and *AUR1-C*). The transformation was made by lithium acetate and PEG method according to MatchMaker protocol (Clontech). The selection in yeast for pGBKT7 and pGADT7 is prototroph for tryptophan and leucine, respectively in synthetic dextrose minus tryptophan or leucine (SDO, Single Drop Out). In the case of co-transformation, the selection was made in synthetic dextrose minus tryptophan and leucine (Double Drop Out, DDO). Synthetic dextrose lacking tryptophan and leucine, but with X-α-Gal and Aurobasidin (DDO/X/A), is suitable to test 2 reporter genes (*AUR1-C* and *MEL1*, respectively). QDO is SD lacking tryptophan, leucine, adenine, and histidine, testing 2 reporters (*ADE2* and *HIS3*). QDO/X/A is the most stringent medium to test for protein interactions, lacking tryptophan, leucine, adenine, and histidine but with aurobasidin and X-α-Gal. All plasmids were tested for auto activation of the system, which were carried out in SDO/X/A (synthetic dextrose minus tryptophan or leucine added with aurobasidin and X-α-Gal). Toxicity was also evaluated in SDO (synthetic dextrose minus tryptophan or leucine).

Protein interaction tests were conducted according to MatchMaker Yeast Two-Hybrid System protocol (Clontech). In brief: combinations of bait and pray plasmids were introduced in Y2HGold and plated on DDO, where all transformations should yield colonies and DDO/X/A in which only proteins that interact produced blue colonies. Transformants that grew on DDO/X/A were transferred to QDO/X/A. In all experiments, the positive and negative controls provided by MatchMaker Yeast Two-Hybrid System (Clontech) were used.

### qPCR

Total RNAs were obtained from various strains incubated overnight in liquid YEPD under 150 rpm agitation at 30 °C. RNA extraction was performed as described before^13^. cDNA synthesis was done with the RevertAid H minus First Strand cDNA synthesis kit (Thermo Scientific) with Oligo dT and random hexamer primers from 5 μg of total RNA. Real time PCR amplifications were made from diluted cDNA templates (1:10) with 800 ηM target primers, 300 ηM GPDH1 (Glyceraldehyde-3-phosphate dehydrogenase) internal control primers, and 1X Syber Green (Evagreen®). Quantification of the transcript levels was performed in StepOne thermo cycler (Applied Biosystems) using the 2^ΔΔCT^ method normalizing against GPDH1, as previously described^86^. An analysis of variance was performed by Tukey’s multiple comparison test using GraphPad Prism 7.0 software, and *p* values lower than 0.05 were considered statistically significant.

### Transcriptional profile: RNA extraction, RNAseq, and quantification analysis

All RNAseq experiments were performed in triplicates. Total RNA was extracted as described above from H99 and CNU123 strains induced in liquid YEPD for 2 h at 30 °C. RNA quantification, purity, and quality were evaluated in Nanodrop spectrophotometer (Thermo Scientific, Waltham, MA, USA) and RNA Nano 6000 Assay Kit of the Bioanalyzer 2100 system (Agilent Technologies, Santa Clara, CA, USA). Libraries were made from 4 µg of total RNA according to instructions of the Illumina TruSeq Stranded mRNA Sample Prep LS Protocol. Library quantifications were performed by qPCR using the KAPA Illumina qPCR Quantification Kit. Quantification and validation of the libraries were done by quantitative PCR, and they were diluted at the working concentration (2 ηM); libraries were denatured with sodium hydroxide solution at 0.1 N, then diluted to 20 ρM, and loaded into HiiSeq sequencer with the v4 sequencing kit (2 × 100 cycles).

The RNA-seq fastq data were applied to the software Trimmomatic^87^ to remove any Illumina adapters and reads with low quality. The FASTQC (https://www.bioinformatics.babraham.ac.uk/projects/fastqc) was performed to verify the overall quality of the sequencing. The reads were aligned to a reference genome of *C*. *neoformas* var. *grubii* H99 presented at the EnsemblFungi database (https://fungi.ensembl.org/Cryptococcus_neoformans_var_grubii_h99_gca_000149245). The counting table was created by the HTseq-count^88^. The statistical analysis was performed using the R/Bioconductor package DESeq. 2^89^ with the method of shrink log fold changes association with condition presented at the package apeglm^90^. The genes with a false discover rate (FDR), adjusted p-value below 0.05, and log2 fold change above 1.0 were considered as differentially expressed. The gene enrichment analysis was performed by the R/Bioconductor package GOstats^91^ to retrieve the most significant Gene Ontology (GO) categories, and the package Pathview^92^ was used to create the KEGG pathways. The protein-protein interaction network was created using the STRING database^93^ and the web tool cytoscape.js (http://js.cytoscape.org).

## Supplementary information

Supplementary information

Supplementary Dataset 1

## Data Availability

All relevant data are within the paper and its Supporting Information files.
