# Supplementary material for: The regulation of the sulfur amino acid biosynthetic pathway in Cryptococcus neoformans: the relationship of Cys3, Calcineurin, and Gpp2 phosphatases
Source: Sci Rep. 2019 Aug 15;9:11923. doi: 10.1038/s41598-019-48433-5 (PMC6695392; doi:10.1038/s41598-019-48433-5)
Supplement: Supplementary file 1 — Supplementary information [file 41598_2019_48433_MOESM1_ESM.pdf]

# The regulation of the sulfur amino acid biosynthetic pathway in *Cryptococcus neoformans*: the relationship of Cys3, Calcineurin, and Gpp2 phosphatases.

Amanda Teixeira de Melo[1*]; Kevin Felipe Martho[1*]; Thiago Nunes Roberto[1]; Erika S. Nishiduka[2]; Joel Machado Junior[1]; Otávio J. B. Brustolini[3]; Alexandre K. Tashima[2]; Ana Tereza Vasconcelos[3]; Marcelo A. Vallim[1], Renata C. Pascon[1&]

[1] Universidade Federal de São Paulo, Campus Diadema, SP, Brazil

[2] Departamento de Bioquímica, Escola Paulista de Medicina, Universidade Federal de São Paulo Campus São Paulo, SP, Brazil

[3] Laboratório Nacional de Computação Científica- LNCC, Labinfo- Laboratório de Bioinformática, Petrópolis, Rio de Janeiro, Brazil

* These authors contributed equally to this work.

[&] corresponding author: renata.pascon@gmail.com

Supplementary Figure 1: Cys3 conserved domains.

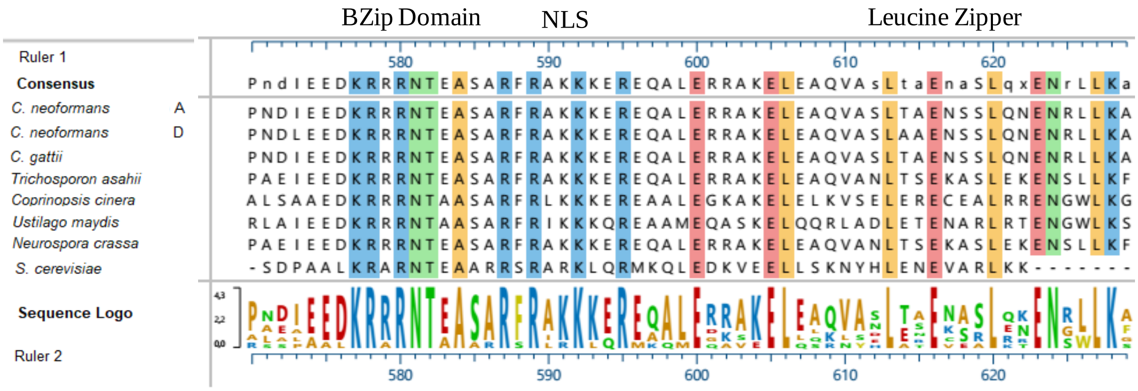

Supplementary Figure 2: Diagnostic PCR (a) and southern blot (b) confirming the deletion of *CYS*3 gene.

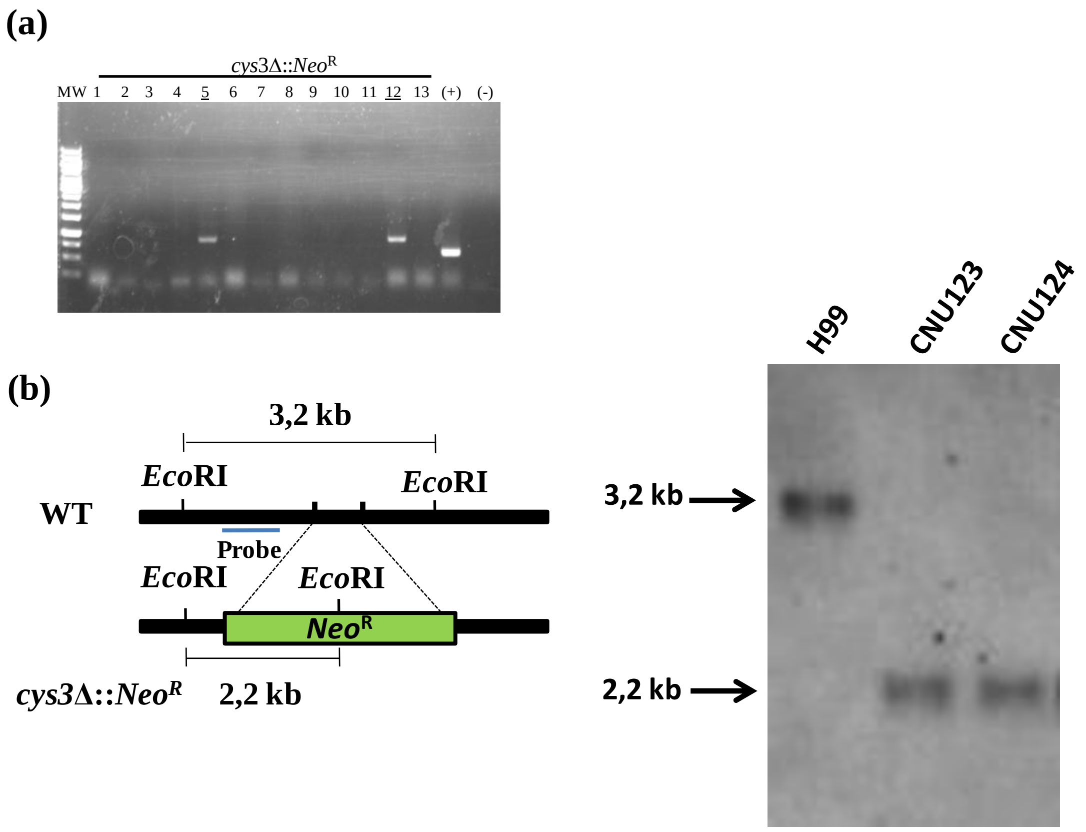

Supplementary Figure 3: *CYS*3expression levels in wild type (H99) and complemented strains.

**(B)**

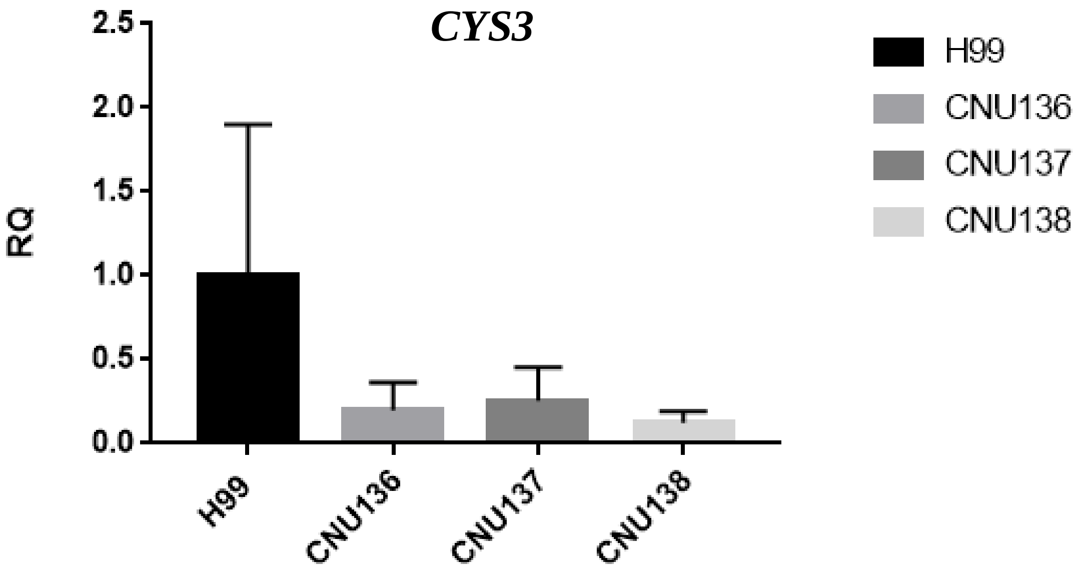

Supplementary Table 1: Strains used in this work

| **Strains** | **Strains background** | **Genotype** | **Source** |
|---|---|---|---|
| HB101 | | *Escherichia coli, F- mcrB mrr hsdS20(rB- mB-) recA13 leuB6 ara-14 proA2 lacY1 galK2 xyl-5 mtl-1 rpsL20(SmR) glnV44 λ-* | Promega (Wisconsin, USA) |
| DH5α | | *Escherichia coli* | Our collection |
| H99 | | *C. neoformans* serotype A, wild type | Our collection |
| JEC21 | | *C. neoformans* serotype D, wild type | Our collection |
| CNU080 | H99 | Gfp:Cys3 | This work |
| CNU119 | CNU080 | *cna1*Δ::*Nat*$^{R}$ | This work |
| CNU121 | CNU080 | *cnb1*Δ::*Nat*$^{R}$ | This work |
| CNU123 | H99 | *cys*-3Δ::*Neo*$^{R}$ | This work |
| CNU124 | H99 | *cys*-3Δ::*Neo*$^{R}$ | This work |
| CNU125 | CNU080 | *gpp2*Δ::*Nat*$^{R}$ | This work |
| CNU136 | H99 | *cys-3*Δ::*CYS3::Hph*$^{R}$ | This work |
| CNU137 | H99 | *cys-3*Δ::*CYS3::Hph*$^{R}$ | This work |
| CNU138 | H99 | *cys-3*Δ::*CYS3::Hph*$^{R}$ | This work |
| CNU145 | JEC21 | Gal7*::CYS-3i*.1 | This work |
| CNU146 | JEC21 | Gal7*::CYS-3i*.2 | This work |
| CNU147 | JEC21 | Gal7*::CYS-3i*.3 | This work |
| Y2HGold | - | *Ade2, His3, Mel1 Aur1-A* | Takara Clontech |
| YL001 | Y2HGold | pGBKT7:*Cnb1* +pGADT7:*Cys-3 #1* | This work |
| YL002 | Y2HGold | pGBKT7:*Cnb1* + pGADT7:*Gpp2* | This work |
| YL003 | Y2HGold | pGBKT7:*Cna1*-ΔC + pGADT7:*Cnb1* | This work |
| YL004 | Y2HGold | pGBKT7:*Cna1*-ΔC + pGADT7:*Cys3* | This work |
| YL005 | Y2HGold | pGBKT7:*Gpp2* + pGADT7:*Cys3* | This work |
| YL006 | Y2HGold | pGBKT7:*Cna1*-ΔC + pGADT7:*Gpp2* | This work |
| YL007 | Y2HGold | pGBKT7-53 + pGADT7-T | This work |
| YL008 | Y2HGold | pGBKT7-53 + pGADT7-T | This work |
| YL009 | Y2HGold | pGBKT7-Lam + pGADT7-T | This work |
| YL0010 | Y2HGold | pGBKT7-Lam + pGADT7-T | This work |
| YL0011 | Y2HGold | pGBKT7:Gpp2 + pGADT7:Cys-3 #1 | This work |

Supplementary Table 2: Plasmids used and constructed in this work.

| **Name** | **Insert** | **Use** | **Source** |
|---|---|---|---|
| pIBB103 | No insert | RNAi | Skowyra & Doering, 2012 |
| pZPHyg | *Hph*$^R$/*Amp*$^R$ | Hygromycin B resistance | Idnurm et al., 2004 |
| pZPNeo | *Neo*$^R$/*Kan*$^R$ | Geneticin resistance | Idnurm et al., 2004 |
| pCN50 | *Neo*$^{R/}$*KanR* | GFP fusion | Gift (Dr. Andrew Alspaugh) |
| pGBKT7 | Empty vector | Bait | Takara Clontech |
| pGADT7 | Empty vector | Prey | Takara Clontech |
| pGBKT7-53 | Murine p53 insert | Positive control | Takara Clontech |
| pGBKT7-Lam | Human lamin C | Negative control | Takara Clontech |
| pGADT7-T | SV40 large T-antigen | Negative and positive controls | Takara Clontech |
| pRCP059 | pIBB103::*cys-3i.1*, Amp$^R$ | RNAi in *CYS-3* | This work |
| pRCP060 | pIBB103::*cys-3i.2*, Amp$^R$ | RNAi in *CYS-3* | This work |
| pRCP061 | pIBB103::*cys-3i.2*, Amp$^R$ | RNAi in *CYS-3* | This work |
| pRCP063 | pGBKT7:*Cys-3* | Bait | This work |
| pRCP065 | pGADT7:Cna1 | Prey | This work |
| pRCP066 | pGBKT7:*Cys-3*AD1 #3 | Bait | This work |
| pRCP067 | pGBKT7:*Cys-3*AD1 #6 | Bait | This work |
| pRCP068 | pGBKT7:*Cys-3*AD1 #8 | Bait | This work |
| pRCP069 | pGBKT7:*Cys-3*AD2 #10 | Bait | This work |
| pRCP071 | pGBKT7:G*pp2 #2* | Bait | This work |
| pRCP073 | pGBKT7:C*na1* #11 | Bait | This work |
| pRCP074 | pGBKT7:*Cys-3*AD3 clone #1 | Bait | This work |
| pRCP075 | pGBKT7:*Cys-3*AD3 clone #4 | Prey | This work |
| pRCP076 | pGADT7:C*ys-3*#1 | Prey | This work |
| pRCP080 | pGBKT7:*Cys-3* A (5´) #1 | Bait | This work |
| pRCP081 | pGBKT7:*Cys-3* A (5´) #2 | Bait | This work |
| pRCP086 | pGBKT7:*Cys-3* B (3´) #7 | Bait | This work |
| pRCP087 | pGBKT7:*Cys-3* B (3´) #8 | Bait | This work |
| pRCP088 | pGADT7:*Gpp2* #10 | Prey | This work |
| pRCP089 | pGBKT7:C*nb1*#1 | Bait | This work |
| pRCP092 | pGBKT7:*Cna1*-ΔC #1 | Bait | This work |
| pRCP096 | pGADT7:*Cnb1* #11 | Prey | This work |

Supplementary table 3: Primers used in this work.

| Primer Code | Sequence | F/R | Use |
|---|---|---|---|
| MAV162 | GACTCACCTTGGGCAGTGGG | R | Homologous integration confirmation (actin promoter) |
| MAV231 | TAATACGACTCACTATAGG | F | T7 promoter, pGBKT7 or pGADT7 insert confirmation |
| MAV240 | AGTATGACTCCACACATGGTCG | F | qPCR *GPDH* |
| MAV241 | AGACAAACATCGGAGCATCAGC | R | qPCR *GPDH* |
| PRCP211 | CCCGAACATCGCCTCGCTC | R | Double joint PCR Hph cassete construction |
| PRCP212 | ATCCCCATGTGTATCACTGGC | F | Double joint PCR Hph cassete construction |
| PRCP213 | ACGACGGGCGTTCCTTGCG | F | Double joint PCR Neo cassete |
| PRCP214 | TCGCTTGGTGGTCGAATGGG | R | Double joint PCR Neo cassete |
| PRCP246 | GGGCCATTCGTCTACCACTG | F | qPCR *CYS3*(CNAG_04798) |
| PRCP247 | TTCGTCGCTTGTCCTCCTCG | R | qPCR *CYS3*(CNAG_04798) |
| PRCP257 | GGATATTCCAAAAGCCCGCTAC | F | *CYS-3* deletion |
| PRCP258 | CTCCAGCTCACATCCTCGCAGGCTGCTGGTGACTATG TCG | R | *CYS-3* deletion |
| PRCP259 | CGACATAGTCACCAGCAGCCTGCGAGGATGTGAGCT GGAG | F | *CYS-3* deletion |
| PRCP260 | CCCACGACATTTGTCACCGAAGAGATGTAGAAACTA GCTTCC | R | *CYS-3* deletion |
| PRCP261 | GGAAGCTAGTTTCTACATCTCTTCGGTGACAAATGTC GTGGG | F | *CYS-3* deletion |
| PRCP262 | TTCGGGTTTGTTGCTGCTGC | R | *CYS-3* deletion |
| PRCP263 | CTGTCCAGGGTTAGCGCCC | F | *CYS-3* homologous integration confirmation |
| PRCP303 | GCTACAGGATCCATGTTCCCCGACCTTCCC | F | *GFP:CYS-3* ( |
| PRCP304 | GCTACAGGATCCTTCACTAAAAGACGTGC | R | *GFP:CYS-3* |
| PRCP305 | GAGCTGTACGAGCTCGGATCCATGTTCCCCGACCTTC CC | F | *GFP:CYS-3* infusion method |
| PRCP306 | GCGGCCGTTACTAGTGGATCCTTCACTAAAAGACGT GC | R | *GFP:CYS-3* infusion method |
| PRCP307 | GCTACAACTAGTCAGTTTCATCCGCCAGCAAAG | F | *CYS-3* RNAi construct |
| PRCP308 | GCTACAACTAGTGCACTGACAGTGCTCGCAC | R | *CYS-3* RNAi construct |
| PRCP309 | CTTCAAGTTCCTTGGCTCGTC | F | RT-PCR CYS3 JEC21 |
| PRCP310 | GACCTCGAAGAGGACAAGCG | R | RT-PCR CYS3 JEC21 |
| PRCP338 | CATGGAGGCCAGTGAATTCATGTCCGTCTTCACCAA GTC | F | pGADT7:*Gpp2* Cloning construct |
| PRCP339 | CTGCAGCTCGAGCTCGATGGATCCTTACTCGTCCTGA GACATTTC | R | pGADT7:*Gpp2* Cloning construct |

| | | | |
|---|---|---|---|
| PRCP340 | CATGGAGGCCAGTGAATTCATGGCTTCCCCAGCCAC TCAG | F | pGADT7:*Cna1* Cloning construct |
| PRCP341 | GCTCGAGCTCGATGGATCCTCACTCGCCTTGACCGCC CTTC | R | pGADT7:*Cna1* Cloning construct |
| PRCP346 | CCATGGAGGCCGAATTCATGTCCGTCTTCACCAAGTC | F | pGBKT7:*Gpp2* Cloning construct |
| PRCP347 | GCAGGTCGACGGATCCTTACTCGTCCTGAGACATTTC | R | pGBKT7:*Gpp2* Cloning construct |
| PRCP348 | CCATGGAGGCCGAATTCATGGCTTCCCCAGCCACTC AG | F | pGBKT7:*Cna1* Cloning construct |
| PRCP349 | GCAGGTCGACGGATCCTCACTCGCCTTGACCGCCCTT C | R | pGBKT7:*Cna1* Cloning construct |
| PRCP383 | gctacaACTAGTTCAATACCATTCCGGGTGAC | F | RNAi *CYS3* (RNAi2) JEC 21 |
| PRCP384 | gctacaACTAGTCTGGTGTAGCTACGGTGGC | R | RNAi *CYS3* (RNAi2) JEC 21 |
| PRCP385 | gctacaACTAGTGCATTCTGGGCCCACCCTCG | F | RNAi *CYS3* (RNAi3) JEC 21 |
| PRCP386 | gctacaACTAGTGTGCTCGCACTTGACACGCC | R | RNAi *CYS3* (RNAi3) JEC 21 |
| PRCP387 | gctacaACTAGTCGCAGGATGAAGAGAAGAAG | F | RNAi *CYS3* (RNAi4) JEC 21 |
| PRCP388 | gctacaACTAGTGCTTATTGTTTGAGCAGCGG | R | RNAi *CYS3* (RNAi4) JEC 21 |
| PRCP393 | GGAATTCTCTCCGAATATCC | F | *CNA1* deletion construct |
| PRCP394 | CTCCAGCTCACATCCTCGCAGACGGAAATTGACTGTT TGGTG | R | *CNA1* deletion construct |
| PRCP395 | CACCAAACAGTCAATTTCCGTCTGCGAGGATGTGAG CTGGAG | F | *CNA1* deletion construct |
| PRCP396 | GTTCGAAACCAGCATCTACTCGAAGAGATGTAGAAA CTAGCTTCC | R | *CNA1* deletion construct |
| PRCP397 | GGAAGCTAGTTTCTACATCTCTTCGAGTAGATGCTGG TTTCGAAC | F | *CNA1* deletion construct |
| PRCP398 | TGATGTTTGATCCGGAAATGG | R | *CNA1* deletion construct |
| PRCP399 | CCGCGAGTTTTCCCTTATCG | F | *CNA1* deletion construct confirmation |
| PRCP400 | CCGCAAATACCAAACATCATC | F | *GPP2*deletion construct |
| PRCP401 | CTCCAGCTCACATCCTCGCATTTGGATAATTACTTGG GGGTC | R | *GPP2*deletion construct |
| PRCP402 | GACCCCCAAGTAATTATCCAAATGCGAGGATGTGAG CTGGAG | F | *GPP2*deletion construct |
| PRCP403 | ATCGCTACATTTAACACATTGAAGAGATGTAGAAAC TAGCTTCC | R | *GPP2*deletion construct |
| PRCP404 | GGAAGCTAGTTTCTACATCTCTTCAATGTGTTAAATG TAGCGAT | F | *GPP2*deletion construct |
| PRCP405 | GCCCACGTATTGTACTTCGTTC | R | *GPP2* deletion cassete construction confirmation |
| PRCP407 | GTTCATGTACAGCTACACTTTGC | F | *CNB1* deletion construct |
| PRCP408 | CTCCAGCTCACATCCTCGCATGCAATAAGGCGGTATT GATGATG | R | *CNB1* deletion construct |
| PRCP409 | CATCATCAATACCGCCTTATTGCATGCGAGGATGTGA GCTGGAG | F | *CNB1* deletion construct |
| PRCP410 | GTATATGGGGTAGGAATGAGAAAGAAGAGATGTAG AAACTAGCTTCC | R | *CNB1* deletion construct |
| PRCP411 | GGAAGCTAGTTTCTACATCTCTTCTTTCTCATTCCTAC CCCATATAC | F | *CNB1* deletion construct |

| | | | |
|---|---|---|---|
| PRCP412 | CGCAGGAAGTCGTTGGGAATC | R | *CNB1* deletion construct |
| PRCP413 | CCCAGACGAGGGTATGTCAG | F | *CNB1* deletion cassete construction confirmation |
| PRCP418 | CCATGGAGGCCGAATTCATGGGTGCCGCTGAATCC | F | pGBKT7:*Cnb1* Cloning costruct |
| PRCP419 | GCAGGTCGACGGATCCTCAGAAGAGATCTTCGAGGG | R | pGBKT7:*Cnb1* Cloning costruct |
| PRCP420 | CATGGAGGCCAGTGAATTCATGGGTGCCGCTGAATCC | F | pGADT7:*Cnb1* Cloning costruct |
| PRCP421 | TCGAGCTCGATGGATCCTCAGAAGAGATCTTCGAGG | R | pGADT7:*Cnb1* Cloning costruct |
| PRCP422 | GCAGGTCGACGGATCCAGCATTAAGAGGGAACTC | R | pGBKT7:*Cna1* Cloning construct (ΔC - terminal) |
| PRCP423 | TCGAGCTCGATGGATCCAGCATTAAGAGGGAACTC | R | pGADT7:*Cna1* Cloning construct (ΔC - terminal) |
| PRCP424 | GATCTTGTGGCTGGTATCACC | F | qPCR *SUL1*(CNAG_00077) |
| PRCP425 | GGTTGAGGGATGAACTCAACG | R | qPCR *SUL1*(CNAG_00077) |

Supplementary Table 4: Down regulated DEGs (Differential Expressed Genes) in the *cys*3Δ mutant.

| ID | Basemean | log2FoldChange | adj. p-value | Gene Description |
|---|---|---|---|---|
| CNAG_05114 | 52,23551559 | -1,094845891 | 3,104E-05 | peroxisomal copper amine oxidase |
| CNAG_05310 | 22,23102951 | -1,021385431 | 0,006762669 | nipsnap family protein |
| CNAG_06863 | 114,4442596 | -1,089749694 | 2,55465E-09 | hypothetical protein |
| CNAG_02270 | 215,9503174 | -1,266851664 | 2,10282E-18 | homoserine O-acetyltransferase |
| CNAG_06557 | 18,07603645 | -1,414185882 | 0,000613258 | membrane protein |
| CNAG_03168 | 2722,432861 | -1,741452336 | 2.38299020450812e-61 | sulfite reductase (NADPH) flavoprotein alpha-component |
| CNAG_04215 | 3465,645752 | -4,473069668 | 0 | sulfate adenylyltransferase |
| CNAG_04901 | 266,8918152 | -1,399561167 | 1,78137E-23 | hypothetical protein |
| CNAG_04798 | 2197,965576 | -1,729887843 | 3.098518093913e-65 | regulatory protein Cys-3 |
| CNAG_01981 | 1883,844482 | -2,158324957 | 9.39235390749535e-77 | sulfide:quinone oxidoreductase |
| CNAG_06448 | 71,79237366 | -1,025355101 | 4,20247E-06 | Cystathionine gamma-lyase |

Supplementary Table 5: Up regulated DEGs (Differential Expressed Genes) in the *cys*3Δ mutant.

| ID | Basemean | log2FoldChange | adj. p-value | Gene Description |
|---|---|---|---|---|
| CNAG_00149 | 532,1651 | 1,272289515 | 0,000936935 | NADH dehydrogenase (ubiquinone) 1 alpha subcomplex 4 |
| CNAG_00654 | 256,39584 | 1,452364683 | 1,26951E-20 | sulfiredoxin |
| CNAG_06758 | 161,76404 | 1,26185739 | 1,05448E-14 | efflux protein |
| CNAG_03650 | 24,887167 | 1,561773181 | 2,11206E-05 | hypothetical protein |
| CNAG_03719 | 428,95734 | 1,229260564 | 7,06869E-24 | hypothetical protein |
| CNAG_03772 | 55,320614 | 1,279858828 | 8,60074E-07 | high-affinity glucose transporter |
| CNAG_02893 | 207,54306 | 1,570221066 | 8,10318E-06 | hypothetical protein, hypothetical protein, variant |
| CNAG_02796 | 947,76563 | 1,059674501 | 1,80263E-13 | 3-deoxy-7-phosphoheptulonate synthase |
| CNAG_02758 | 28,581167 | 1,391358852 | 3,51531E-05 | NADH:flavin oxidoreductase/NADH oxidase |
| CNAG_04951 | 4864,0635 | 1,152758241 | 4,73314E-21 | 3-deoxy-7-phosphoheptulonate synthase |
| CNAG_07797 | 468,11264 | 1,561634064 | 2,35154E-23 | transcriptional regulator, transcriptional regulator, variant |
| CNAG_04974 | 476,68604 | 1,005774617 | 2,24534E-16 | hypothetical protein |
| CNAG_07803 | 109,2313 | 1,040782452 | 1,06511E-08 | hypothetical protein |
| CNAG_05302 | 321,3024 | 1,402369261 | 2,63014E-17 | Amine oxidase |
| CNAG_06823 | 11,318757 | 1,153782845 | 0,024684601 | hypothetical protein |
| CNAG_06805 | 16,979317 | 1,08817625 | 0,010241591 | hypothetical protein |
| CNAG_01354 | 431,22189 | 1,721194863 | 7,3064E-29 | hypothetical protein |
| CNAG_00992 | 3711,0142 | 1,080840945 | 2,39499E-22 | homocitrate synthase, mitochondrial |

| | | | | |
|---|---|---|---|---|
| CNAG_02540 | 638,58307 | 1,436732769 | 2,70567E-22 | hypothetical protein |
| CNAG_02508 | 522,67163 | 1,459818125 | 7,17241E-21 | hypothetical protein |
| CNAG_02143 | 164,31216 | 1,211883307 | 1,30724E-11 | hypothetical protein, hypothetical protein, variant |
| CNAG_03386 | 1297,6168 | 1,057737112 | 8,16204E-15 | solute carrier family 25 (mitochondrial carnitine/acylcarnitine transporter), member 20/29 |
| CNAG_03415 | 208,43896 | 1,241953135 | 2,53052E-12 | hypothetical protein |
| CNAG_07733 | 12,166297 | 1,294737816 | 0,01505156 | hypothetical protein |
| CNAG_03461 | 345,07373 | 1,199350595 | 2,73597E-22 | hypothetical protein, hypothetical protein, variant |
| CNAG_04108 | 1550,3248 | 1,061349869 | 7,1222E-17 | pyruvate dehydrogenase kinase |
| CNAG_04307 | 317,06973 | 1,096140981 | 3,07264E-14 | Urate oxidase |
| CNAG_04347 | 1918,1469 | 1,303316712 | 7,83678E-25 | Aspartate kinase |
| CNAG_01542 | 85,9244 | 1,691048861 | 6,7606E-12 | taurine catabolism dioxygenase TauD |
| CNAG_01603 | 1743,1785 | 2,541198969 | 3.77322340285078e-76 | hypothetical protein |
| CNAG_01668 | 21,797033 | 1,022958636 | 0,005652909 | hypothetical protein |
| CNAG_01953 | 134,16966 | 1,168879151 | 5,37179E-10 | hypothetical protein |
| CNAG_06018 | 375,41934 | 1,014905691 | 2,24919E-18 | aldehyde dehydrogenase (NAD), aldehyde dehydrogenase (NAD), variant |
| CNAG_06374 | 716,2464 | 1,188407779 | 0,002930961 | malate dehydrogenase (oxaloacetate-decarboxylating)(NADP) |
| CNAG_05333 | 52,835644 | 2,750291824 | 3,35017E-19 | hypothetical protein |
| CNAG_05425 | 5745,1738 | 1,203320026 | 3,04738E-27 | asparagine synthase (glutamine-hydrolyzing) |
