# Supplementary material for: The regulation of the sulfur amino acid biosynthetic pathway in Cryptococcus neoformans: the relationship of Cys3, Calcineurin, and Gpp2 phosphatases
Source: Sci Rep. 2019 Aug 15;9:11923. doi: 10.1038/s41598-019-48433-5 (PMC6695392; doi:10.1038/s41598-019-48433-5)

The regulation of the sulfur amino acid biosynthetic pathway in *Cryptococcus neoformans*: the relationship of Cys3, Calcineurin, and Gpp2 phosphatases.

Amanda Teixeira de Melo[1*]; Kevin Felipe Martho[1*]; Thiago Nunes Roberto[1]; Erika S. Nishiduka[2]; Joel Machado Junior[1]; Otávio J. B. Brustolini[3]; Alexandre K. Tashima[2]; Ana Tereza Vasconcelos[3]; Marcelo A. Vallim[1], Renata C. Pascon[1&]

[1] Universidade Federal de São Paulo, Campus Diadema, SP, Brazil

[2] Departamento de Bioquímica, Escola Paulista de Medicina, Universidade Federal de São Paulo Campus São Paulo, SP, Brazil

[3] Laboratório Nacional de Computação Científica- LNCC, Labinfo- Laboratório de Bioinformática, Petrópolis, Rio de Janeiro, Brazil

* These authors contributed equally to this work.

[&] corresponding author: renata.pascon@gmail.com

Supplementary data: original western blot images.

Figure 5c

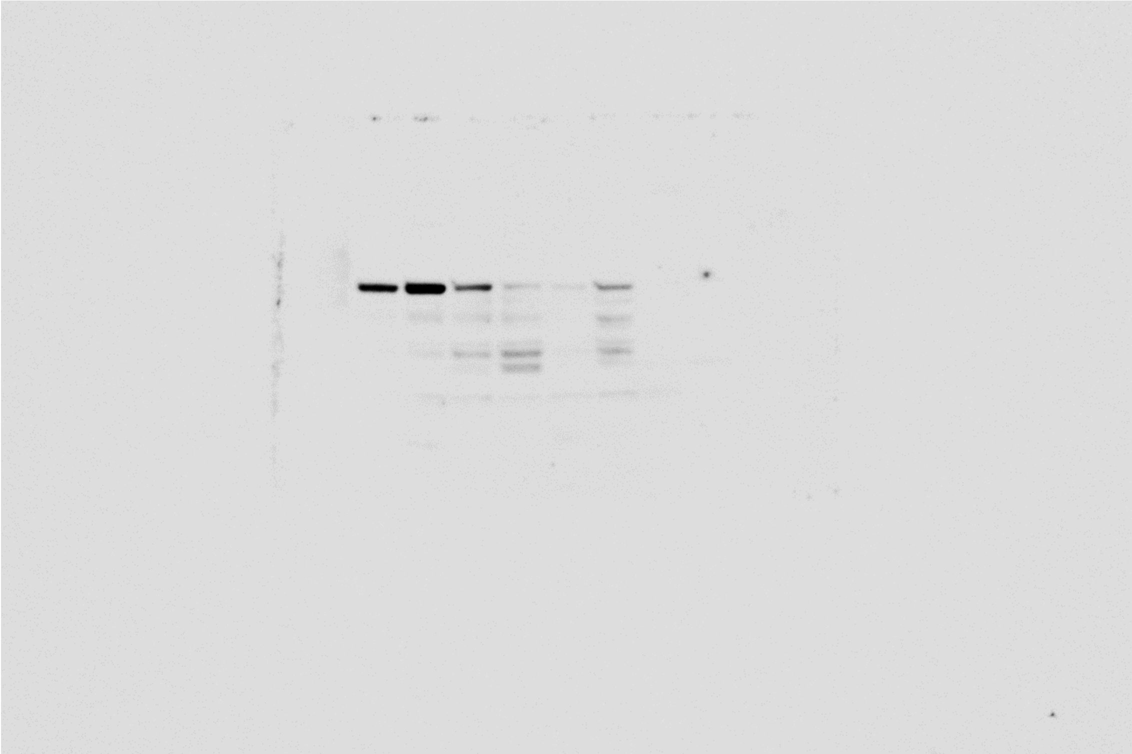

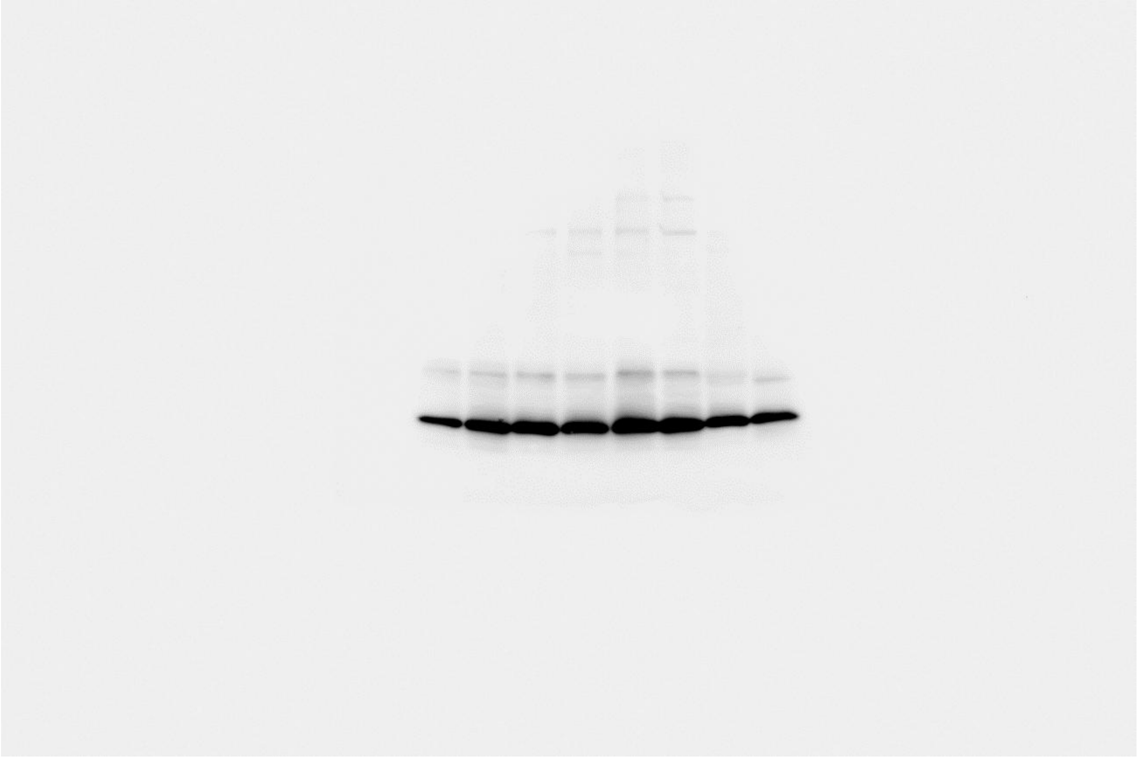

Figure 7c

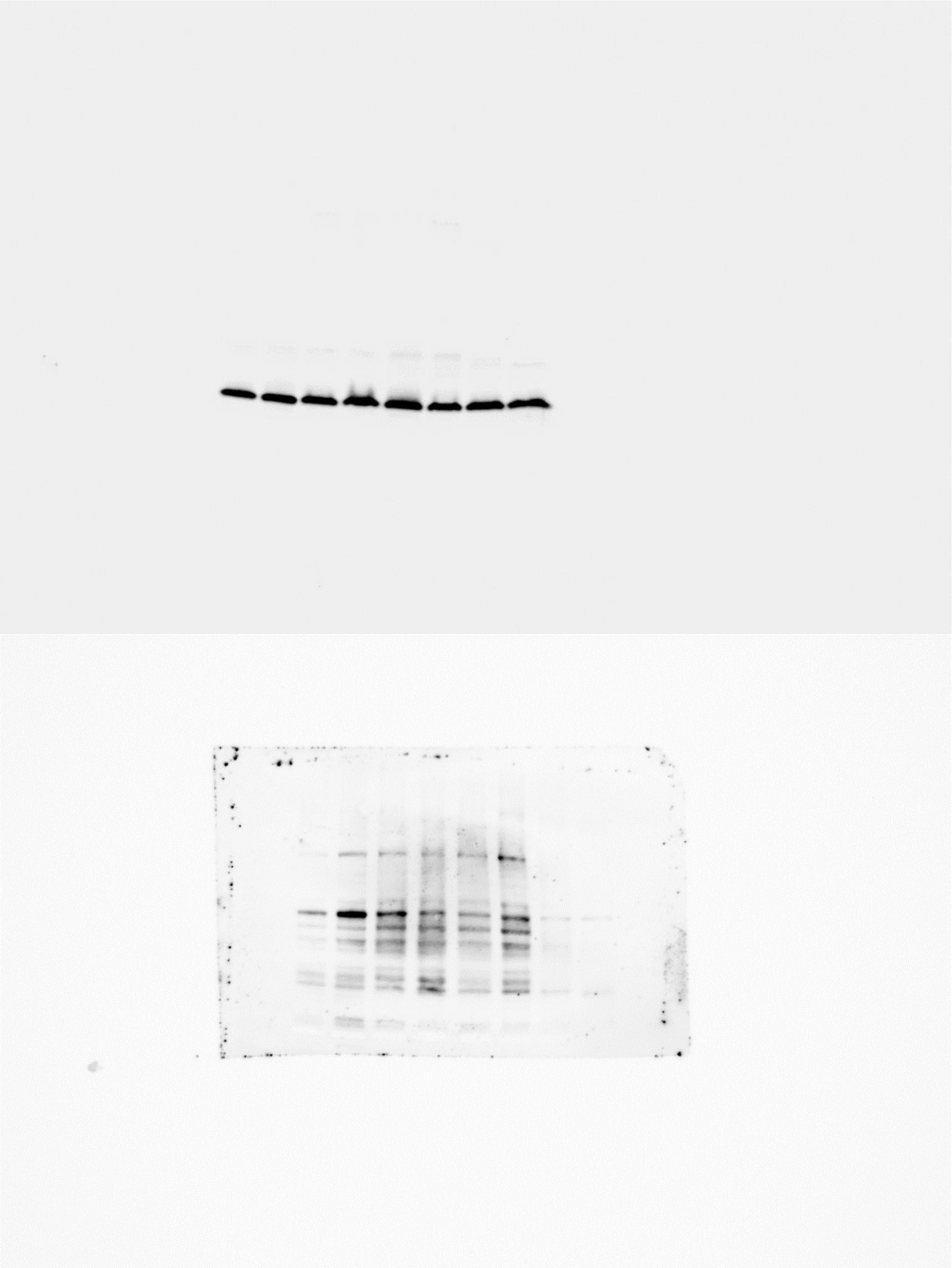

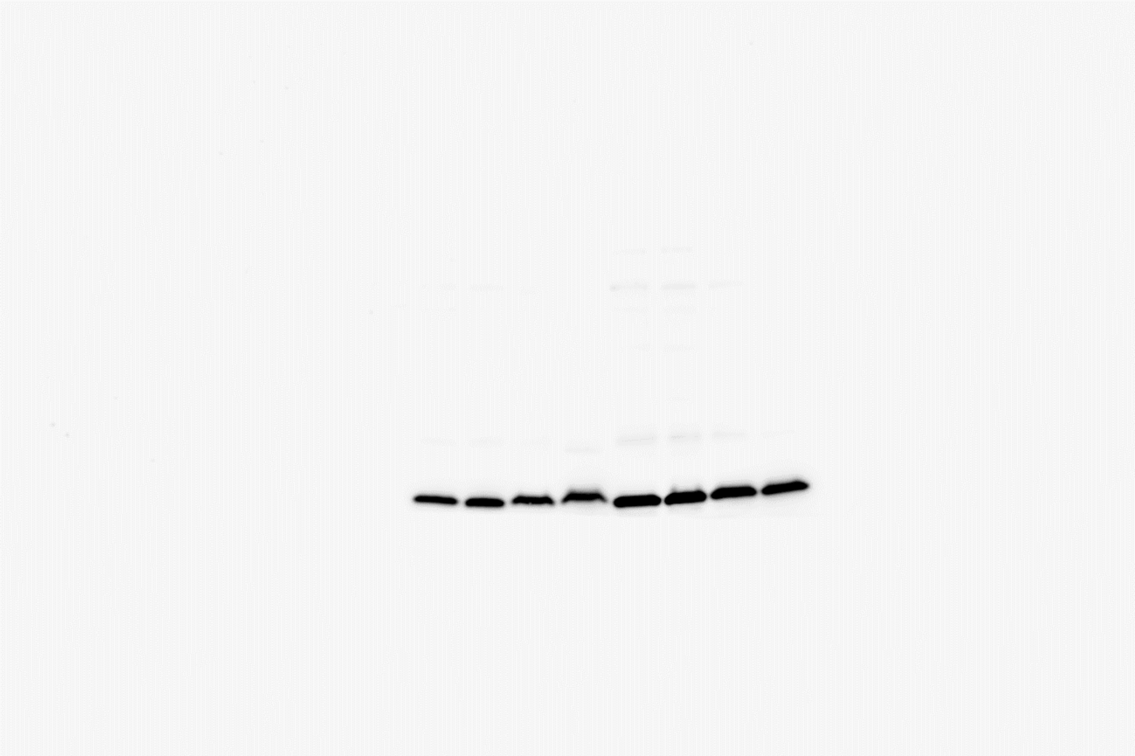

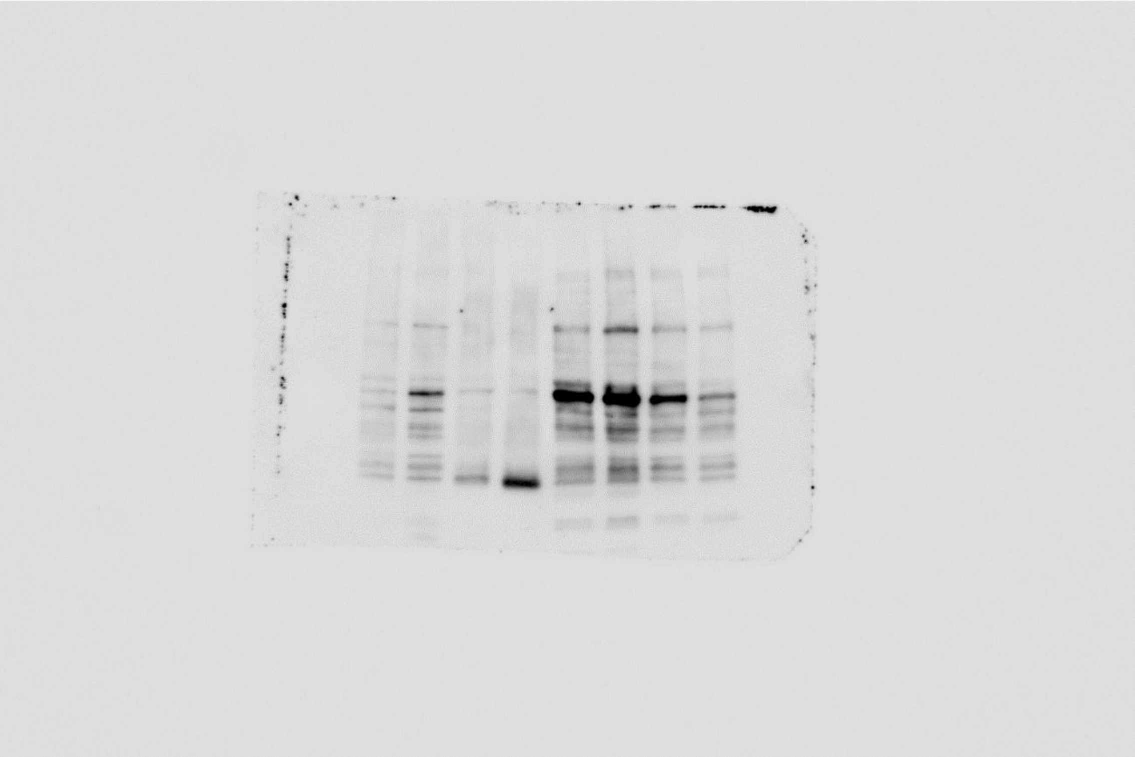

Supplement: Supplementary file 2 — Supplementary Dataset 1 [file 41598_2019_48433_MOESM2_ESM.pdf]
